# Comparing benthic biogeochemistry at a sandy and a muddy site in the Celtic Sea using a model and observations

**DOI:** 10.1007/s10533-017-0367-0

**Published:** 2017-09-07

**Authors:** J. N. Aldridge, G. Lessin, L. O. Amoudry, N. Hicks, T. Hull, J. K. Klar, V. Kitidis, C. L. McNeill, J. Ingels, E. R. Parker, B. Silburn, T. Silva, D. B. Sivyer, H. E. K. Smith, S. Widdicombe, E. M. S. Woodward, J. van der Molen, L. Garcia, S. Kröger

**Affiliations:** 1 Centre for Environment, Fisheries and Aquaculture Science, Lowestoft, NR33 0HT UK; 2 Plymouth Marine Laboratory, Prospect Place, The Hoe, Plymouth, PL1 3DH UK; 3 Scottish Association for Marine Science, Scottish Marine Institute, Oban, Argyll, PA37 1QA UK; 4 Ocean and Earth Science, National Oceanography Centre, University of Southampton, Southampton, SO14 3ZH UK; 5 National Oceanography Centre, Joseph Proudman Building, 6 Brownlow Street, Liverpool, L3 5DA UK; 6 Coastal and Marine Laboratory, Florida State University, 3618 Coastal Highway 98, St Teresa, 32358 FL USA; 7 LEGOS, University of Toulouse, IRD, CNES, CNRS, UPS, 14 avenue Edouard Belin, 31400 Toulouse, France

**Keywords:** Biogeochemistry, Modelling, Celtic Sea, Benthic, Permeable sediments

## Abstract

Results from a 1D setup of the European Regional Seas Ecosystem Model (ERSEM) biogeochemical model were compared with new observations collected under the UK Shelf Seas Biogeochemistry (SSB) programme to assess model performance and clarify elements of shelf-sea benthic biogeochemistry and carbon cycling. Observations from two contrasting sites (muddy and sandy) in the Celtic Sea in otherwise comparable hydrographic conditions were considered, with the focus on the benthic system. A standard model parameterisation with site-specific light and nutrient adjustments was used, along with modifications to the within-seabed diffusivity to accommodate the modelling of permeable (sandy) sediments. Differences between modelled and observed quantities of organic carbon in the bed were interpreted to suggest that a large part (>90%) of the observed benthic organic carbon is biologically relatively inactive. Evidence on the rate at which this inactive fraction is produced will constitute important information to quantify offshore carbon sequestration. Total oxygen uptake and oxic layer depths were within the range of the measured values. Modelled depth average pore water concentrations of ammonium, phosphate and silicate were typically 5–20% of observed values at the muddy site due to an underestimate of concentrations associated with the deeper sediment layers. Model agreement for these nutrients was better at the sandy site, which had lower pore water concentrations, especially deeper in the sediment. Comparison of pore water nitrate with observations had added uncertainty, as the results from process studies at the sites indicated the dominance of the anammox pathway for nitrogen removal; a pathway that is not included in the model. Macrofaunal biomasses were overestimated, although a model run with increased macrofaunal background mortality rates decreased macrofaunal biomass and improved agreement with observations. The decrease in macrofaunal biomass was compensated by an increase in meiofaunal biomass such that total oxygen demand remained within the observed range. The permeable sediment modification reproduced some of the observed behaviour of oxygen penetration depth at the sandy site. It is suggested that future development in ERSEM benthic modelling should focus on: (1) mixing and degradation rates of benthic organic matter, (2) validation of benthic faunal biomass against large scale spatial datasets, (3) incorporation of anammox in the benthic nitrogen cycle, and (4) further developments to represent permeable sediment processes.

**Electronic supplementary material:**

The online version of this article (doi:10.1007/s10533-017-0367-0) contains supplementary material, which is available to authorized users.

## Introduction

Important gaps remain in understanding coupled pelagic-benthic biogeochemical processes. These include quantification of carbon supply into the bed, incorporation of material by biological and physical processes and the fate of carbon during the annual cycle. Observations of marine systems are always incomplete, spatially constrained, and many key processes, such as carbon cycling and coupling to biological processes, are hard to observe. Models play an important role in interpreting and complementing observations. Consequently, there is a need to closely link knowledge and understanding from observational programmes to developments in modelling and vice versa. An important aspect of this is the comparison of observations with model ‘predictions’ (so called model validation) in order to gauge model performance and identify areas where model improvements should be focussed. Validation is a key step in the cycle of model development and important in assessing the reliability, as well as potential limitations, of model results.

Though biogeochemical models have been commonly used to examine biogeochemical processing of carbon and nutrients in shelf seas (e.g. Moll and Radach 2003; Vichi et al. 2004b; Blackford et al. 2004; Lenhart et al. 2010), the emphasis has generally been on pelagic processes rather than the benthic system, benthic-pelagic links and processing of organic material in the benthic system. Conversely, although many models of sediment diagenesis have been developed (see Paraska et al. 2014), most have limited representation of benthic fauna and their interaction with geochemical cycles, and rely on specification of pelagic forcing as a boundary condition. Also, few have been coupled to biogeochemical models of the pelagic system. Where fully coupled models have been developed, validation of the benthic component of coupled pelagic-benthic biogeochemical models at locations on the continental shelf appears relatively infrequent (examples include Blackford 1997; Soetaert et al. 2001; Capet et al. 2016) compared to validation of the pelagic system, highlighting an important gap in biogeochemical modelling of shelf seas.

We specifically aim to address the paucity of validation and model-data comparison studies for the benthic system. To that end, we use a recent version of the European Regional Seas Ecosystem Model (ERSEM) (Butenschön et al. 2016), a coupled pelagic and benthic biogeochemical model, and compare model results with field observations from the Celtic Sea collected under the UK Shelf Seas Biogeochemistry (SSB) programme. Comparisons are undertaken both with a ‘standard’ parameter set and with a modified set, where adjustments were made to improve the fit to observations. The aim of the paper is not to provide a re-parameterisation of the benthic model, as that should be done with a much wider set of data to avoid over tuning at a limited set of locations. Nevertheless, one of the main outputs is a list of recommendations to consider in future developments of the benthic model.

An initial step was also made towards including within the model the effects of pore water flows associated with permeable sediments (sands and gravels). In permeable sediments, transport is driven mainly by pore water flow and exchange with the overlying water column (Huettel et al. 1996; Marinelli et al. 1998; Huettel and Webster 2000; Huettel et al. 2014) rather than molecular diffusion, the dominant transport mechanism in muddier non-permeable substrates. Laboratory and field observations suggest that rates of oxygen uptake (Janssen et al. 2005; Cook et al. 2007), organic matter breakdown, and denitrification (Cook et al. 2006) can be higher in permeable sediments than non-permeable sediments. Permeable sediment processes have been modelled and compared with laboratory experiments (Cook et al. 2006; Janssen et al. 2012; Kessler et al. 2012), but a key challenge is to evaluate the significance of these observational results at shelf sea scales. One approach is to incorporate these processes into appropriate models applicable to shelf wide studies, such as used here.

## Study region

**Fig. 1:**
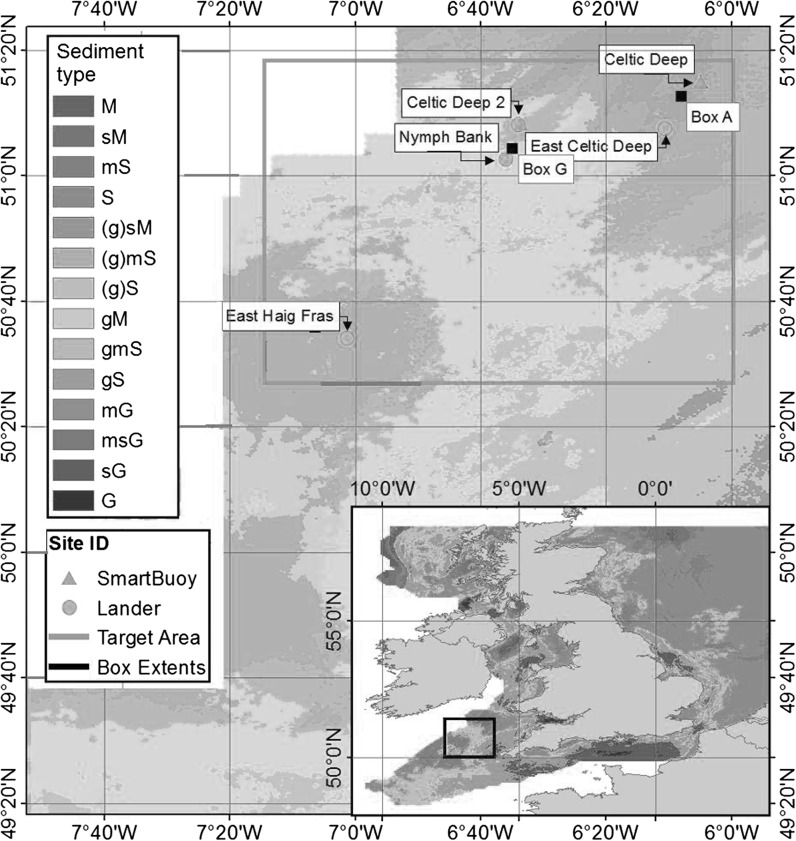
Location map of the SSB Celtic Sea study sites superimposed on sediment type information based on Folk classification (Folk 1954). Note at the Celtic Deep 2 site there are superimposed lander and Cefas SmartBuoy deployments, Box A, G etc. are synonymous with ‘site A’ and ‘site G’ in the text

The shelf seas biogeochemistry (SSB) program included pelagic and benthic studies during the years 2014–2015. The five main SSB Celtic Sea benthic study sites, labelled Box A, Box I, Box H, and Box G, together with the ‘Candyfloss’ site (Fig. 1) covered a range of bed sediments with differing proportions of sand to mud, but with broadly similar physical and water column characteristics. A summary of the sites and the benthic observational program can be found in Thompson et al. (2017). For the purpose of our analysis we focus on the extremes: two sites that differ most in sediment type—box A (most muddy sediment) and box G (most sandy sediment). A summary of key site specific physical parameters is given in Table 1.

**Table 1:** Site specific bulk parameters taken from measurements reported in Thompson et al. (2017)

Site	Water depth (m)	% fine (<63 μm)	Median grain diameter (mm)	Sediment type (Folk class)	Porosity	Permeability^1^ (m^2^)
Box A	103	54	0.057	Sandy mud (sM)	0.68	–
Box G	98	13	0.46	Sand (S)	0.44	5.0 × 10^−11^

Values are means over depth range 0–5 cm of sediment from all samples collected at the sites

^1^Permeability from Jahnke et al. (2005)

## Methods

### Summary of observations

Observed quantities used for model comparison were as follows: (a) Water column temperature, oxygen and nitrate, ammonium, phosphorus, silicate and chlorophyll concentrations (mmol m^−3^). (b) Total (benthic) oxygen uptake (TOU, mmol O_2_ m^−2^ day^−1^). (c) Oxygen penetration depth (OPD, m), defined in the model as the depth at which the free oxygen concentration becomes zero. (d) Particulate organic carbon (POC) as profiles (mg C m^−3^) and depth-integrated (mg C m^−2^); depth-integrated benthic particulate organic nitrogen (PON, mg N m^−2^). (e) Pore water ammonium nitrate, phosphate, and silicate concentration (mmol [N, P, Si] m^−3^). (f) Depth-integrated macrofaunal, meiofaunal and bacterial biomass density (g C m^−2^). This is summarised in Table 2. For locations see Fig. 1.

**Table 2:** Summary of observational data used in model-data comparison

Quantity	Units	Where	How	DOI
Surface temperature	^o^C	Celtic Deep 2 (adjacent to site G)	SmartBuoy moored thermometer 1 m below sea surface.	10.14466/CefasDataHub.39
Bottom temperature	^o^C	Celtic Deep 2 (CD2), East Celtic Deep (ECD), Nymph Bank (NB) East Haig Fras (EHF)	Seabed landers thermometer approximately 1 m above seabed. Deployments at different sites were not in general simultaneous.	10.14466/CefasDataHub.40 10.14466/CefasDataHub.41 10.14466/CefasDataHub.42 10.14466/CefasDataHub.38
Surface chlorophyll	mg m^−3^	Celtic Deep 2 (adjacent to site G)	SmartBuoy moored fluorimeter, 1 m below sea surface.	10.14466/CefasDataHub.39
Pelagic: Nitrate, Ammonium, Phosphate, Silicate	mmol m^−3^	Site A and G	Water samples collected on CTD castes.	10.5285/2eb8d803-8823-1e6f-e053-6c86abc052a6 10.5285/2eb8d803-8822-1e6f-e053-6c86abc052a6
Benthic organic Carbon Nitrogen	g C m^−3^ mmol N m^−3^	Site A and G	Site A cores, 1 cm slices 0–25 cm. Site G cores, bulk 0–5 cm and 5–10 cm only.	10.5285/47110529-757c-40b5-e053-6c86abc0eddc
Benthic Oxygen uptake (TOU)	mmol O_2_ m^−2^ day^−1^	Site A and G	(a) Oxygen decrease in water above core. (b) Bulk slurry (see text).	10.5285/47110529-757b-40b5-e053-6c86abc0eddc
Oxygen penetration depth (OPD)	cm	Site A and G	Oxygen probe	10.5285/47110529-757d-40b5-e053-6c86abc0eddc
Pore water: Nitrate, Ammonium, Phosphate, Silicate	mmol m^−3^	Site A and G	Sampling tubes inserted into cores	10.5285/487b5547-e454-76b1-e053-6c86abc000a3
Macro and Meiofaunal biomass	g C m^−2^	Site A and G	Sieving and counting of grab samples	Not yet available
Bacterial biomass	g C m^−2^	Site A and G	See main text	Not yet available

Surface temperature, oxygen and night-time fluorescence were measured by the Cefas SmartBuoy mooring (http://www.cefas.co.uk/monitoring) at the Celtic Deep 2 site and averaged from half-hourly observations to daily values for model comparison. Daytime fluorescence measurements near the sea surface were discarded as they can be affected by background light levels and fluorescence quenching. Night-time values were converted to chlorophyll concentration by calibration with water samples collected at the site.

Bottom oxygen and temperature were obtained from measurements at the Celtic Deep 2 (CD2), East Celtic Deep (ECD), Nymph Bank (NB) and East Haig Fras (EHF) sites. The latter site, although approximately 70 km from the sites clustered around the Celtic Deep, had the longest temperature record. Where other deployments overlapped in time, temperatures here were found to be essentially identical (see "Pelagic" section) to those at the other sites and so the EHF data was included in the model comparison.

Pelagic nutrient concentrations profiles were obtained from analyses of water samples collected during Conductivity Temperature Density (CTD) casts from eight cruises (three in 2014 and five in 2015).

Benthic process measurements were taken at the study sites Box A, I, H, G, during four benthic cruises in March/April 2014, March 2015, May 2015, and August 2015. Details of these measurements are described elsewhere (Thompson et al. 2017; Hicks et al. 2017; Silburn et al. 2017; Kitidis et al. 2017) and only a summary is given here. The multi-partner nature of the SSB benthic program meant that for some quantities, independent measurements were taken by more than one group enabling an assessment of the observational uncertainty.

Total oxygen uptake (TOU) measurements were obtained from shipboard analysis of cores. Three independent measurements were available, here denoted by the lead investigators initials as NH, HS, VK respectively. One method (NH) measured oxygen decrease in the overlying water immediately after collection, one (HS) after aeration for 18 hours. The third (VK), used mass spectrometry to get total oxygen content (water + sediment) averaged over four replicate cores on collection and averaged over four cores after a 40-minute incubation. The difference between the before and after results yielded a single (replicate averaged) oxygen consumption estimate. Measurements were taken under conditions without interstitial flows which may add to uncertainty in values obtained for permeable sediments.

Two independent measurements of oxygen penetration depth were obtained (denoted NH, BS) both using glass oxygen electrodes to determine the point at which free oxygen reached a constant, small value (see Hicks et al. 2017; Silburn et al. 2017).

Measurements of benthic particulate organic carbon (POC) and nitrogen (PON) at site A were from cores sliced at 0.5 cm intervals (top 1–2 cm) and 1 cm intervals (2–25 cm). Site G observations were bulk values over 0–5 and 5–10 cm. Organic carbon and nitrogen content was measured by an elemental analyser to obtain values with units of weight C, N per weight of dry sediment. For comparison with the model these were converted to weight per cubic metre using the observed dry sediment weight per unit volume measured on the same core.

Two independent measurements of nutrient pore water profiles (denoted in plots as DS, JK) were obtained at site A and one at site G (DS) (Kitidis et al. 2017; Klar et al. 2017).

**Fig. 2:**
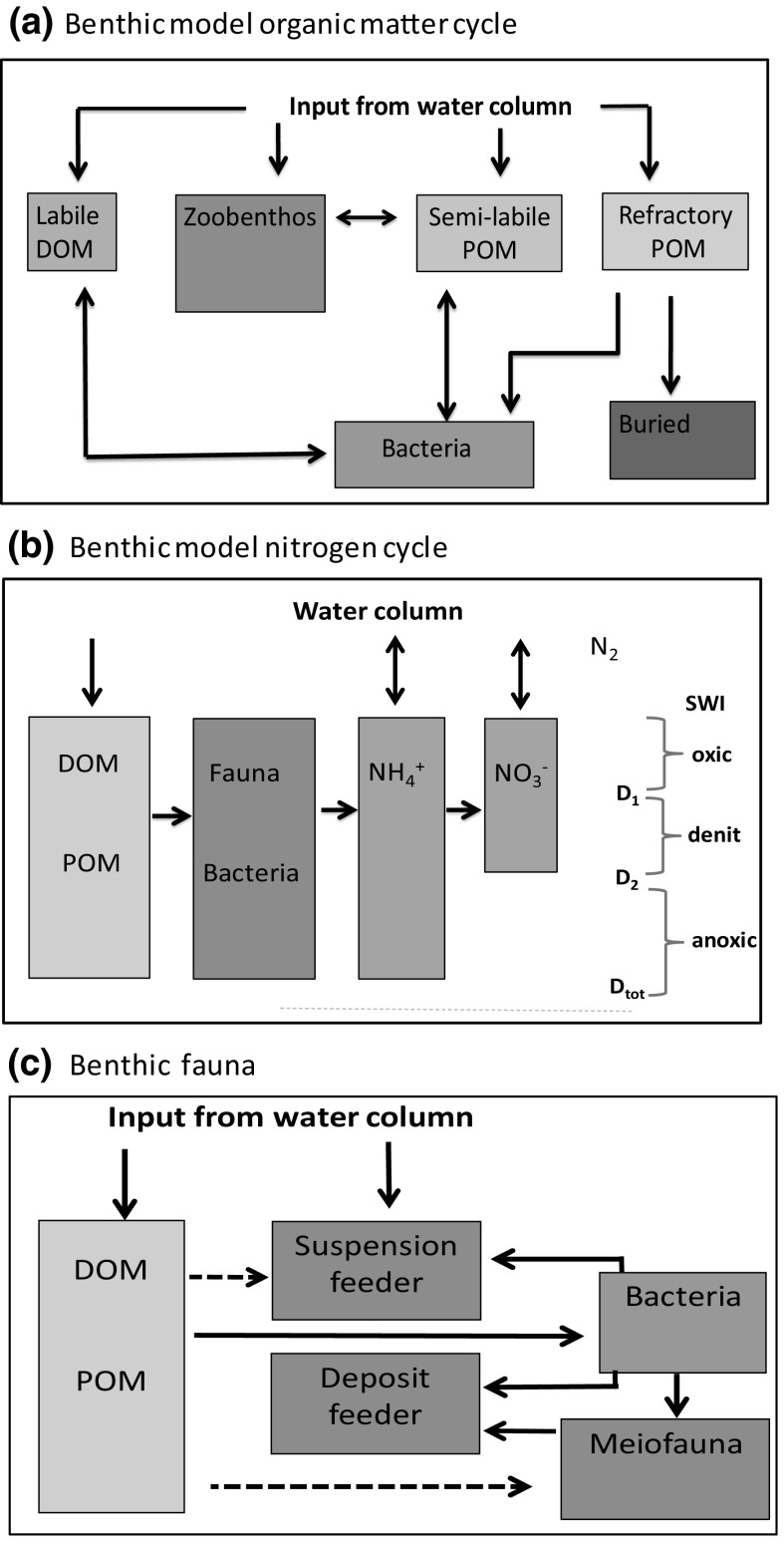
**a** ERSEM 15.06 model benthic organic matter classes and relationships. **b** Simplified schematic of main ERSEM 15.06 benthic nitrogen cycle. D1 is the oxygen penetration depth, D2 is depth at which the nitrate concentration becomes zero. **c** Benthic food web. *Dotted arrows* indicate less preferred paths. *SWI* sediment water interface. *Note* faunal groups also excrete material to the POM/DOM pool (not shown)

The macro benthic infauna was sampled using a 0.08 m^3^ NIOZ box corer with 5 replicates obtained at each of the four sites for four different cruises. The 80 sediment samples were sieved with a 1 mm mesh and all specimens analysed and dry blotted wet weight was measured individually. The ERSEM 15.06 model used in this study divides macro fauna into deposit feeder and suspension feeder functional groups (Fig. 2c). Observed faunal species were therefore classified similarly. Wet weight biomass was converted to carbon mass per m^2^. Classification of feeding mode and carbon: wet-weight ratios for observed taxa was done using the PhyloPars algorithm (Bruggeman et al. 2009, available at http://www.ibi.vu.nl/programs/phylopars) in conjunction with the database of Brey et al. (2010). In both cases, the measure of species similarity was based on the WoRMS taxonomy (WoRMS Editorial Board 2017). Note, the procedure that was used assigned species into either deposit or suspension groups even if their primary feeding mode was (say) predation.

For meiofaunal analysis, three cores were taken from the same box cores as used for the macrofaunal measurements. After sieving with a 63 μm mesh, the nematode component only was measured for two of the cruises (April 2014, March 2015) and the wet weight estimated from dimensions (see Thompson et al. 2017). Nematodes comprised on average 85% of numerical abundance at the Celtic Sea sites, and body size covered most of the meiofaunal size range, suggesting that most of the observed meiofaunal biomass is accounted for. Meiofauna data were available for the first two cruises only. For meiofauna nematodes, a single factor of 0.124 g C/g wet weight (Giere 2009) was used to convert from wet weight to carbon weight.

Measured bacterial biomass was derived from the methodology of Main et al. (2015). Phospholipid-derived fatty acids (PLFA) were extracted from 3.0 g freeze dried sediment from the 0-1 cm horizon of each core (3 replicates per site, per season). Bacterial carbon biomass was estimated from the concentration of three PLFA bacterial biomarkers (15:0i, 15:0ai and 16:0i) (Mayor et al. 2012; Moodley et al. 2005), assuming that the PLFAs constitute 10% of the total bacterial PLFA, and applying the conversion factor of 0.056 g C PLFA/g C biomass (see Thompson et al. 2017).

### Model description

#### Overview

The biogeochemical model used was ERSEM 15.06 (Butenschön et al. 2016). Compared to most shelf scale biogeochemical models, ERSEM was developed with a relatively complete benthic description (Ruardij and Van Raaphorst 1995; Ebenhoh et al. 1995). The benthic sub model used in this study implements the dissolved flux model of Kohlmeier (2004) (see also Vichi et al. 2004a). To provide one-dimensional vertical (1DV) fields for physical variables (light, temperature, and water column mixing), ERSEM 15.06 was coupled to the General Ocean Turbulence Model (GOTM) water column physics model (Burchard et al. 2006). For the calculations presented here, GOTM and ERSEM 15.06 was run under the Framework for Aquatic Biogeochemical Models (FABM) (Bruggeman and Bolding 2014).

A very comprehensive description of ERSEM 15.06 is given in Butenschön et al. (2016). However, the most relevant components of the benthic model are described here to help the reader to better understand the comparison with observations presented later. Processing of organic material (OM) is the starting point for most benthic activity and in the model water column detritus and phytoplankton enter the benthic system via: (1) consumption and sub-surface excretion by suspension feeding macrofauna, and (2) by direct settling to the top of the sediment and incorporation into the seabed (Fig. 2a). For the latter pathway, ERSEM 15.06 assigns incoming pelagic material into benthic pools of labile dissolved organic matter (DOM), a semi-labile particulate organic matter (POM) and (semi-) refractory POM using specified ratios (Blackford 1997). The benthic DOM component is assumed to be consumed exclusively by aerobic bacteria and generally represents a small proportion of the benthic carbon content compared with POM. Within the bed, vertical POM profiles are assumed to follow an exponential profile decreasing from the surface and, separately for each of carbon, nitrogen, phosphate and silicate, characterised by a dynamically calculated total quantity in the bed and characteristic penetration depth mainly controlled by modelled bioturbation intensity (Ebenhoh et al. 1995; Butenschön et al. 2016).

Inorganic nutrients in the ERSEM 15.06 benthic model are represented in terms of the total (depth integrated) bed nutrient content (pore water plus adsorbed) for nitrate, ammonium, phosphate and silicate (Kohlmeier 2004; Vichi et al. 2004a). Pore water and adsorbed nutrient phases are assumed to be in instantaneous equilibrium, controlled by a fixed (but possibly layer-dependent) partition coefficient. The total benthic nutrient content is calculated with reference to an assumed vertical bed structure consisting of (1) an oxygenated layer, bounded below by the OPD; (2) a transition layer, bounded below by what we will term the nitrate penetration depth, where nitrate but not free oxygen is present; (3) an anoxic layer. The depth of these layers is determined dynamically, with oxygen penetration depth and nitrate penetration depth defined as the depth where oxygen and nitrate respectively reach zero due to aerobic consumption and denitrification. Pore water nutrient content is calculated based on source and sink terms in each layer and an implicit assumption of diffusive transport within the layer. At each time step, the total nutrient content that would be attained at equilibrium is determined based on the layer source and sinks. The benthic pelagic flux and updated bed nutrient content is calculated assuming relaxation toward the equilibrium value. The nitrogen cycle implemented in the model (Fig. 2b), includes bacterially mediated conversion of dissolved and particulate organic nitrogen (DON, PON) to ammonium, together with nitrification, denitrification, and exchange with the overlying water. Denitrification is assumed to depend on anaerobic bacterial biomass and nitrate concentration; the nitrification flux is modelled as a first order process proportional to depth-averaged ammonium concentration. The anammox (Anaerobic Ammonium Oxidation) pathway to N_2_ is not included. Benthic inorganic phosphate content is determined by production from organic forms by biota, oxygen-dependent adsorption, and exchange with overlying waters. Pore water silicate generation is modelled as a first order process proportional to the concentration of silicate in POM.

The ERSEM 15.06 benthic food web (Fig. 2c) includes three benthic faunal groups distinguished mainly by feeding mode: filter feeders (infaunal and epifaunal), deposit feeders (infaunal) and meiofauna, together with aerobic and anaerobic bacteria. As well as feeding preferences, each group has an associated set of physiological parameters such as maximum uptake rates, background mortality, and traits such as bioturbation potential (Ebenhoh et al. 1995). Within a functional group ERSEM makes no size distinction so that parameter and trait values represent an average over a range of organism sizes.

Coupled 1DV water column models were set up for box A and G from the main SSB Celtic Sea benthic sites (Fig. 1). A hundred vertical layers were used to represent the water column, giving an average vertical resolution of around one metre, with increased resolution at the sea surface (minimum layer thickness 20 cm) and bed (minimum layer thickness 70 cm). Bed porosity was set to measured values at the sites (Table 1; Thompson et al. 2017). Meteorological forcing, covering the period 1995–2015 inclusive, was obtained from the European Centre for Medium Range Weather Forecasting “ERA-Interim” dataset (0.75° horizontal resolution) and linearly interpolated to the site locations. Vertical water-column mixing by tidal currents was included by specifying M2 and S2 current amplitude and phase information at the study sites obtained from a shelf-wide tidal model (Bricheno et al. 2015). The original ERSEM formulation for calculating pelagic oxygen saturation (Anonymous 1964; Baretta and Ruardij 1988) was used in the presented results rather than the alternative formulation (Weiss 1970) as the former gave better agreement for near-surface oxygen measurements in the Celtic Sea. Salinity was not calculated dynamically but fixed at a constant value of 35.2 psu based on average surface salinity measured by the Celtic Deep 2 Cefas SmartBuoy. Temperature and velocity were set to physically reasonable but arbitrary initial values as these are determined subsequently by the applied forcing. To compensate for the absence of horizontal advection of temperature within a one-dimensional model, modelled temperatures were relaxed toward observed bottom measurements from seabed landers. Attempts to simultaneously relax to measured surface and bottom temperatures produced unrealistic temperature profiles and were not used.

Site-specific parameters and initial conditions were set.to reproduce broadly the behaviour observed for pelagic quantities as follows. (1) Water-column nutrient concentrations were set at the start of the simulation to the observed average winter values in 2015–2016 at sites A and G. (2) The timing of the spring bloom was adjusted to fit observations by altering the background (suspended particulate matter) light-extinction coefficient. (3) An external summer flux of nitrate and phosphate into the sea surface was included to maintain phytoplankton production after the spring bloom, with a corresponding removal of nutrients in winter to avoid long term changes in nutrient content. This could be regarded as a compensation for the absence within the one-dimensional model of horizontal advection of nutrients. The seasonal flux was justified on the basis that it is desirable to ensure the benthic system (the focus of this study) is, as far as possible, not compromised by potential deficiencies in the pelagic component. To prevent run-down of total nitrogen content due to denitrification, the benthic denitrification flux was fed back into the sea surface as nitrate. Injection at the surface to some extent mimics atmospheric deposition of nitrate (Prospero et al. 1996) that is not explicitly included in the model.

The combined GOTM-ERSEM 15.06 model was run for a 21-year period from the beginning of 1995 to the end of 2015. Benthic variables were monitored to ensure that by 2014–2015, when results were compared with observations, the model had reached a stable repeating state apart from perturbations due to the meteorological forcing.

#### Permeable sediment modification

The ERSEM benthic formulation assumes that transport within the bed and across the sediment water interface (SWI) is driven by molecular diffusion (possibly enhanced by bio-irrigation). Although appropriate for muddy sediments, in permeable sediments pore water flows will also contribute to transport and exchange. Although pore water flows can be driven by a range of mechanisms (Santos et al. 2012) the focus here is on the interaction of bottom currents with seabed ripples, setting up pressure gradients that drive pore-water flows (Huettel et al. 1998; Huettel and Webster 2000).

The ERSEM 15.06 benthic model divides the bed into oxic and anoxic layers, with a transition layer between the two at the oxygen penetration depth *D*_1_. To fit within the present framework the effect of pore water flows was simulated by enhancing the existing within-bed diffusion coefficient in the top (oxic layer of the bed) as

1 $$K = (K_{0} + K_{{adv}} )I_{{bio}}$$

where *K*_*0* is_ the background (molecular) diffusion coefficient and *K*_*adv*_ is the new contribution that mimics the increased flushing of sediment due to the pore water flow. *I*_*bio*_ is a standard ERSEM bio-irrigation factor (Blackford 1997) accounting for increase in exchange area from biological activity. Observational studies indicate that the depth of pore water flow scales with the ripple dimensions (Huettel and Webster 2000). As, dimensionally, the diffusion coefficient is the product of a velocity scale and length scale, a natural representation for effect of the pore water flow is

2 $$K_{adv} = a_{2} w_{0 } { \hbox{min} }(h, D_{1} )$$

where *w*_*0*_ is a measure of the pore water flow rate (ms^−1^), the length scale is taken as the minimum of the ripple height (*h*) and the oxic layer depth (*D*_1_), and a_2_ = 4.0, is a scaling constant. This constant was set to reproduce observed levels of OPD in the observations. The velocity *w*_*0*_ is taken equal to the Darcy flow velocity which, following Rutherford et al. (1995), is calculated over bedforms as

3 $$w_{0} = \, k \, \Delta P/\left( {\rho \, \nu \, L} \right)$$

where *k* (m^2^) is the permeability, *ρ* is water density (kg m^−3^), *ν* is kinematic viscosity (m^2^ t^−1^), *L* is the ripple wavelength (m) and Δ*P* (kg m^−1^ s^−2^) is the pressure difference across the ripple induced by the near-bed flow. Typically, flow will penetrate the bed to a distance scaling on the ripple height. Based on the experimental work in Janssen et al. (2012) the pressure difference was expressed as

4 $$\Delta P \, = \, a_{1} \rho \, U^{2} (h/L)$$

where *a*_*1*_ = 1.0 is an empirical constant, *ρ* is water density (1010 kg m^−3^), *U* is a near-bed reference velocity and *h/L* is the ripple slope. The reference velocity *U* is taken at 10 cm and is calculated assuming a logarithmic velocity profile

5 $$U = \sqrt {\tau _{b} /\rho }\,\kappa \ln \left( {{\text{z}}_{{\text{r}}} /{\text{z}}_{0} } \right)$$

where *τ*_*b*_, the bed stress, is calculated by the hydrodynamic model, the von Karman constant *κ* = 0.41, the reference height *z*_*r*_ = 0.1 m and the bed roughness *z*_*0*_ = *h/*7.0 where *h* is the ripple height (Soulsby 1997).^1^ The ripple height and wavelength were set provisionally at 3 and 20 cm respectively based on estimates from seabed imagery at site G. Permeability was not measured in the field work. However, Jahnke et al. (2005) report an average permeability for sands of median diameter essentially identical to that at site G (505 µm, site G = 460 µm) and we use their value of *k* = 5.0 × 10^−11^ m^2^ in Eq. (3).

This modification is an attempt to include effects of interstitial flows in a simple way with minimum change to the existing framework. As such it represents only a first step in addressing the potential complexities of interstitial flows.

#### Model run configurations

Model results are shown for site A (mud) with a standard model parameter set (‘Model A’), and for site G with the permeable sediment modification included (‘Model G’) and with the modification switched off (‘Model G0’). A preliminary assessment and sensitivity study of benthic model results suggested some simple parameter adjustments to improve model agreement with observations. The results were shown as an additional site A run (‘Model A1’). In this run the rate of refractory POM breakdown by bacteria and conversion to a semi labile form (Fig. 2a) was increased by a factor of five (from 2 × 10^−6^ to 1 × 10^−5^ m^2^ (g C)^−1^ day^−1^). To maintain a similar refractory POM content to the standard run, the ratio of pelagic detritus going into semi labile and refractory POM was altered from approximately 9:1 to a value 1:1. It is not suggested that this is necessarily closer to reality, the objective was to test the general effect of moving from a system where, after consumption of labile spring bloom material, the bed is becoming depleted of available POM by late winter (as happens with the original parameter setting at this site) to one where a significant residue of semi labile material is present throughout the year. Model A1 also introduced increases in deposit and suspension feeder background mortality rates by a factor of 4 and 2 respectively (from 0.001 to 0.004 day^−1^ and 0.001 to 0.002 day^−1^) to see if model re-parameterisation can better reproduce observed biomass without adversely affecting other model results.

## Results

### Pelagic

Although the focus of the paper is the benthic system, a basic validation of pelagic variables was undertaken to identify factors that might influence the performance of the benthic sub-model. A set of time-depth contour plots of model temperature, nitrate, ammonium, phosphate, silicate, oxygen and chlorophyll for the period 2014–2015 are available in Figures A–F (Online Resource 1). A more detailed discussion of a comparison with observations is given here.

**Fig. 3:**
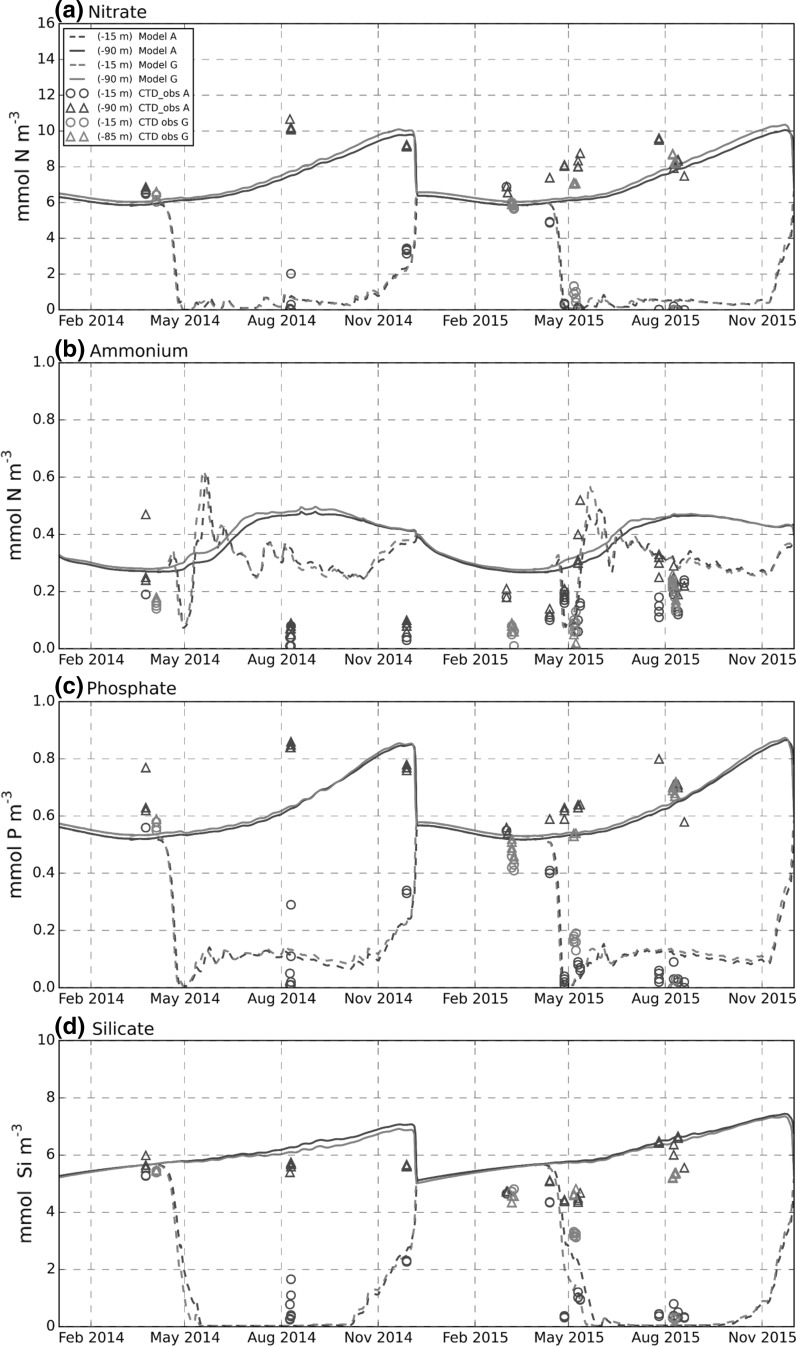
Pelagic nutrients, model-data comparison at sites A and G. Observed nutrients from water samples taken during CTD casts. *Line/symbol colour* indicates site, *Circles* near-surface (−15 m), *triangles* near-bottom values (−85– −90 m). Model, *dashed curves* near-surface values, *solid* curves near-bottom values

The modelled seasonal cycle of bottom and surface nitrate, phosphate and silicate concentrations at site A and G were generally in good agreement with water sample measurements (Fig. 3a, c, d). The agreement with winter concentrations is not surprising since water-column nutrients were set at the start of the model run by reference to the observed winter concentrations from 2010 to 2015, and the model nutrient cycle is a closed system. Nevertheless, the agreement with observed winter concentrations at the end of the run indicated that any shift of nutrients between the water column and the benthic system was small relative to water-column content. The decrease in surface concentrations in May 2015 matched closely with observations. Measured concentrations also broadly supported the increase in bottom nitrate, phosphate and silicate over spring and summer seen in the model. There appeared to be no clear difference between water-column nutrient concentrations observed at site A and G, consistent with model results which were essentially identical at the two sites.

**Fig. 4:**
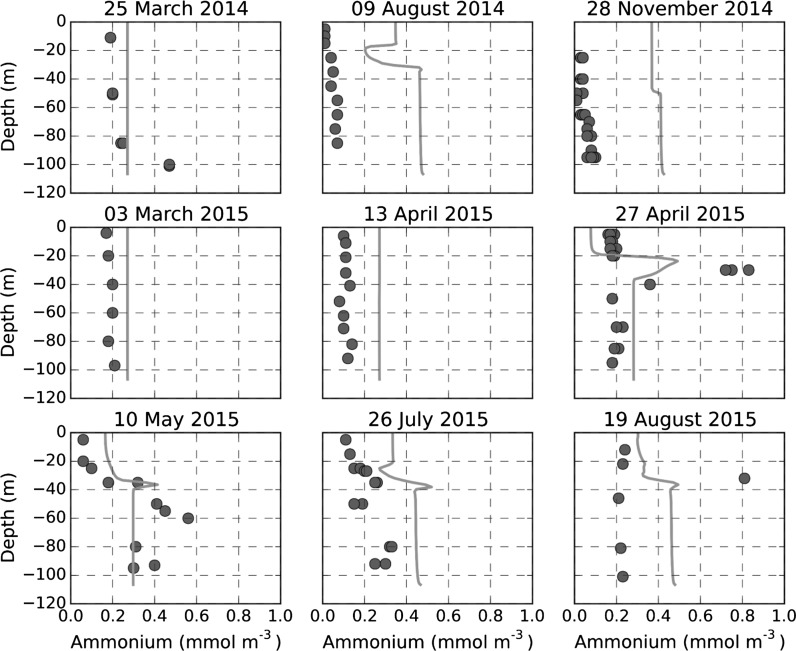
Ammonium water column profiles, model-data comparison near site A. Model (*line*); observations CTD casts (*circles*)

**Fig. 5:**
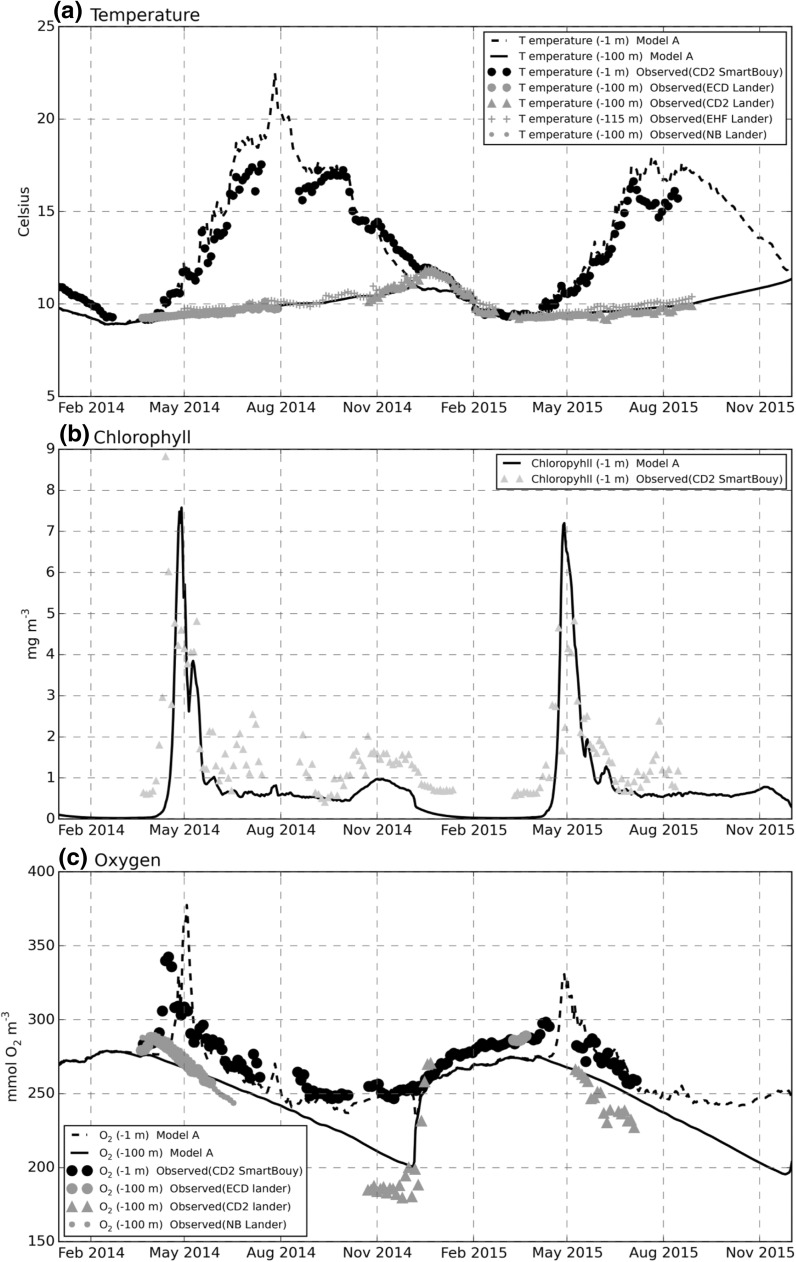
Temperature, chlorophyll, oxygen, model-data comparison for pelagic variables near site A. **a** Temperature (°C), near-surface (−1 m) and near-bottom (−100, −115m). **b** Surface (−1 m) chlorophyll (mg Chl m^−3^). **c** Oxygen concentration (mmol m^−3^), near-surface (−1 m) and near-bottom (−100 m). Model site G values are almost identical to A and not shown. *Surface values* from Cefas SmartBuoy. *Bottom values* from seabed lander deployments. Celtic Deep 2 (CD2); East Celtic Deep (ECD); East Haig Fras (EHF); Nymph Bank (NB). See Fig. 1 for locations

Ammonium concentrations showed a greater difference between modelled and observed values. Observations showed significant inter-annual variability and less distinct separation between surface and bottom values that was not captured by the model (Fig. 3b). Observed and modelled ammonium profiles at site A (Fig. 4) showed reasonable agreement in March 2014, but thereafter observed values in 2014 decreased throughout the water column while model concentrations remained high. In general, measured ammonium concentrations in 2014 were smaller than in 2015 with the model in better agreement with 2015 values. Thus in 2015 although profiles in March and early April were overestimated by the model, the 27th April and 10th May 2015 profiles were in reasonable agreement. By 26th July through to 19th August, observed concentrations had decreased while modelled bottom concentrations increased, then remained constant. Interestingly, observed and modelled profiles both showed a maximum at the transition between the upper and lower mixed layers in April and August 2015, corresponding to the location of a modelled deep chlorophyll maximum (Online Resource 1, Figures C, F), suggesting the ammonium peak is associated with this. Modelled surface temperatures were in good agreement with the Celtic Deep Smart buoy measurements in spring and autumn, but appeared to be overestimated in July and August in both years (Fig. 5a). Observed bottom temperatures showed little spatial variability over scales of 40 km, this being the approximate distance of the East of Haig Fras lander from the other lander measurement. Modelled bottom temperatures were successfully relaxed to observed values leading to a good reproduction of the onset of stratification in 2014 and 2015 and correct bottom temperatures through most of the year. However, remixing of the water column was about a month earlier than observed at the end of 2014 due to too rapid cooling of surface temperature in the model in early winter. Differences between model temperature predictions at site A and G were negligible. Model runs without relaxation to observed values (not shown) underestimated winter temperatures by about 1.5 °C, probably because observed winter temperatures are moderated by advective transports (e.g. an Atlantic influence) that are absent in a 1D water column model.

The modelled chlorophyll was calculated by summing over four phytoplankton groups in the model (Fig. 5b). As described in the "Methods" section, the timing of the spring bloom was calibrated to fit the observed spring bloom at the Celtic Deep SmartBuoy by adjusting the light regime based on the background suspended particulate matter concentration and a seasonal flux of nitrate and phosphate was added to maintain chlorophyll concentrations through the summer. There is still an underestimate (up to a factor of 2) in modelled chlorophyll after the spring bloom, although concentrations are closer to those measured in 2015 when most of the benthic observations were made. An indication of a small autumn bloom in 2014 was reflected in the model results, with the correct timing and duration, but underestimated in magnitude. In the post spring bloom period, model results show highest chlorophyll concentrations at the interface between surface and bottom mixed layers (Online Resource 1, Figure F) suggesting that surface chlorophyll concentrations may represent a less important contribution to total water column productivity during summer.

Modelled surface oxygen concentrations generally showed good agreement with Celtic Deep SmartBuoy data (Fig. 5c), apart from an underestimate of winter values at the start of 2015 after water-column remixing. Observed peak surface-oxygen concentrations associated with the 2014 spring bloom were qualitatively reproduced by the model. In 2015 there was a break in the observations during the spring bloom, so values here could not be confirmed, but predicted post-bloom oxygen concentrations were in good agreement with observations. Bottom oxygen concentrations after the onset of stratification decreased less rapidly in the model than observed, leading to a 10% overestimate in early winter just before water-column remixing.

### Benthic organic material

**Fig. 6:**
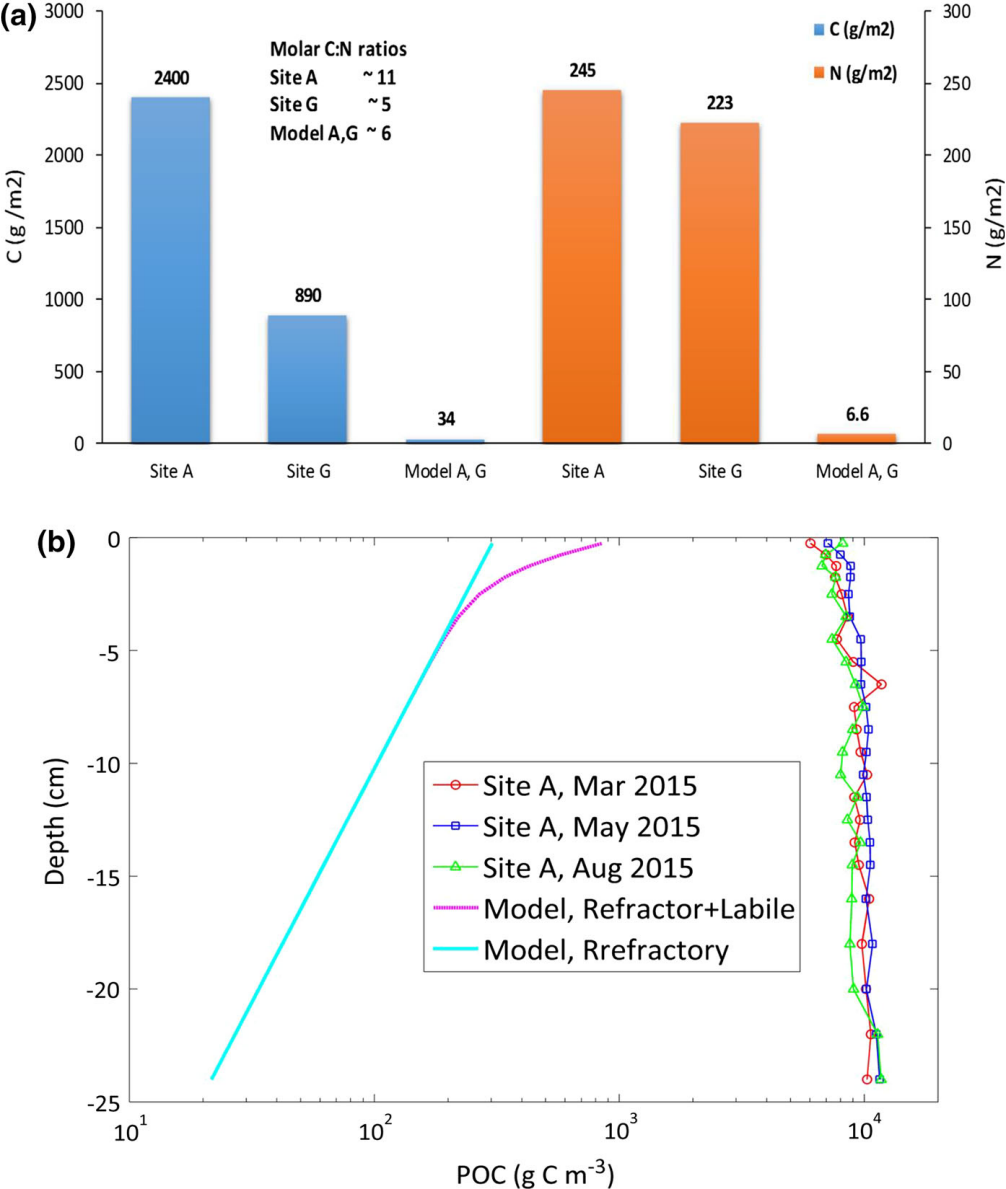
Particulate organic carbon and nitrogen model-data comparison. **a** Total bed inventory (annual average, g C m^−2^, g N m^−2^). Observations, 25 cm deep cores at site A and 10 cm deep cores at G, with site G values scaled to 25 cm assuming uniform values with depth. Model values, sum of semi-labile and refractory concentrations integrated down to 25 cm. **b** POC profiles (g C m^−3^) at site A. Model curves are annual average values for site A. See "Methods" section and Fig. 2 for definition of refractory and semi labile POM in the model

Processing of organic material (OM) is the starting point for most benthic activity (Arndt et al. 2013) and is considered first. Modelled semi-labile plus refractory POC integrated to 25 cm depth was between 1% (muddy site A) to 4% (sandy site G) of the observed value, with modelled PON around 2% (site A and G) of that observed (Fig. 6a). Thus, measured organic material in the bed was significantly larger than the quantity of benthic POM in the model. The amount of buried (biologically inactive) POC in the model (see Fig. 2a) increased from zero at the start of the run to around 6 g C m^−2^ after 21 years (i.e. a burial rate of 0.3 g C m^−2^ year^−1^). The difference in total benthic POM in model results for site A and G was minor (<5%).

Based on the modelled penetration depth and quantity of organic carbon in the bed, the implicit exponential variation with depth of modelled POM can be compared against observations. High resolution depth profiles of organic carbon were not available at site G so results are shown for site A only (Fig. 6b). At the sediment surface, model POC depth concentrations (g C m^−3^) were approximately 10% of the measured value, decreasing to around 1% at 10 cm depth. Semi-labile POC was only present in the top 3–4 cm. Observed POC was relatively uniform down to 25 cm, with a tendency to increase with depth due to decreasing porosity.

### Benthic oxygen dynamics

**Fig. 7:**
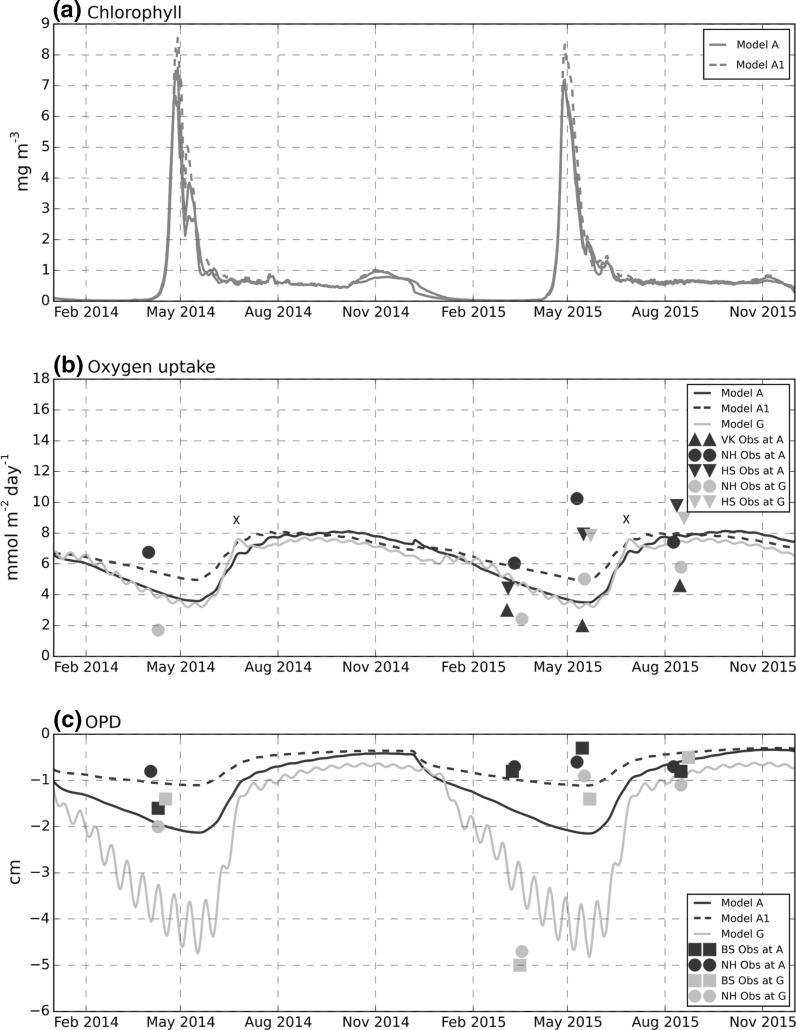
Benthic oxygen, model-data comparison at sites A and G. **a** Water column, near-surface chlorophyll (mg Chl m^−3^) included for temporal reference in interpreting benthic variables. **b** Total (benthic) oxygen uptake (mmol O_2_ m^−2^ day^−1^) plotted as a positive value (NB as a flux into the bed, this is often given a negative value). **c** Oxygen penetration depth (cm), negative from the SWI. *Observational data* NH, HS, VK, BS denotes data from independent measurements of this quantity as described in the "Methods" section. Note dates of observations have been adjusted slightly to avoid overlapping of symbols. Periods of enhanced oxygen consumption in model G marked by X

Modelled benthic total oxygen consumption (TOU) was within the range of observed values (Fig. 7b), although with the standard parameter settings (Model A) it was at the lower end of measured values at the muddy site A. Surface chlorophyll concentration is shown so benthic results can be evaluated with respect to the timing of the spring bloom (Fig. 7a). Chlorophyll concentrations from the three model runs showed very small differences. Modelled TOU increased after the spring bloom, reaching a maximum in July, then decreased from late summer through winter and spring. The lag on oxygen demand following the spring bloom in the model was partly due to the time required for material to settle through the water column and partly due to the time required for bacterial and faunal biomass to increase in response to spring bloom inputs. Observations, although showing differences in magnitude and to some extent temporal trend, consistently yielded higher oxygen uptake in August compared to earlier in the year. The re-parameterisation of benthic POM breakdown (Model A1) had the effect of sustaining oxygen uptake in the later winter and spring, bringing the model values close to the middle of the observed range prior to and during the spring bloom. The permeable sediment modification (Model G) had very little effect on oxygen uptake apart from the modest increases (marked X) just after the spring bloom.

Modelled oxygen penetration depth (OPD) for the muddy site A with original parameter settings (Model A) was generally deeper than observed (1–2 cm modelled, <1 cm observed) (Fig. 7c). An exception was August 2015, where the model value at both sites was in very close agreement with observations. Deepening of the modelled OPD in early winter coincided with the mixing of the water column and an increase in water column oxygen concentrations at a time of low oxygen demand. The re-parametrisation of benthic POM breakdown (Model A1) increased benthic consumption in winter and early spring, giving a shallower OPD and better agreement with observations prior to, and just after, the spring bloom. The two independent measurements of OPD were consistent at both sites. The clearest difference observed between sites A and G was the value of the OPD in March 2015 (site G = 5 cm, site A = 1 cm).

At the sandy site G, a significant difference in OPD prior to the spring bloom was observed in 2014 and 2015 with OPD in March/April 2014 considerably shallower (2 cm) than March 2015 (5 cm). The value of 5 cm measured in 2015 is more typical of values measured in sandy permeable sediments in the North Sea and English Channel (Parker et al. 2011; Defra 2013). The model results however showed little inter-annual variability and, with the specified value of scaling constant (Eq. 2), site G results were much closer to the deeper March 2015 observations. Modelled behaviour appeared to capture the general pattern observed at site G in 2015, with a deep OPD prior to the spring bloom and a subsequent shallowing in response to increased oxygen demand. However, the observed shallowing of the OPD happened earlier compared to the model, which overestimated considerably the OPD in May. Nevertheless, by August 2015 modelled values were close to observations. The effect of the spring neap cycle on near-bed velocities and therefore on predicted pore water flows and OPD was clearly discernible in the model results for site at G.

### Benthic nutrients

**Fig. 8:**
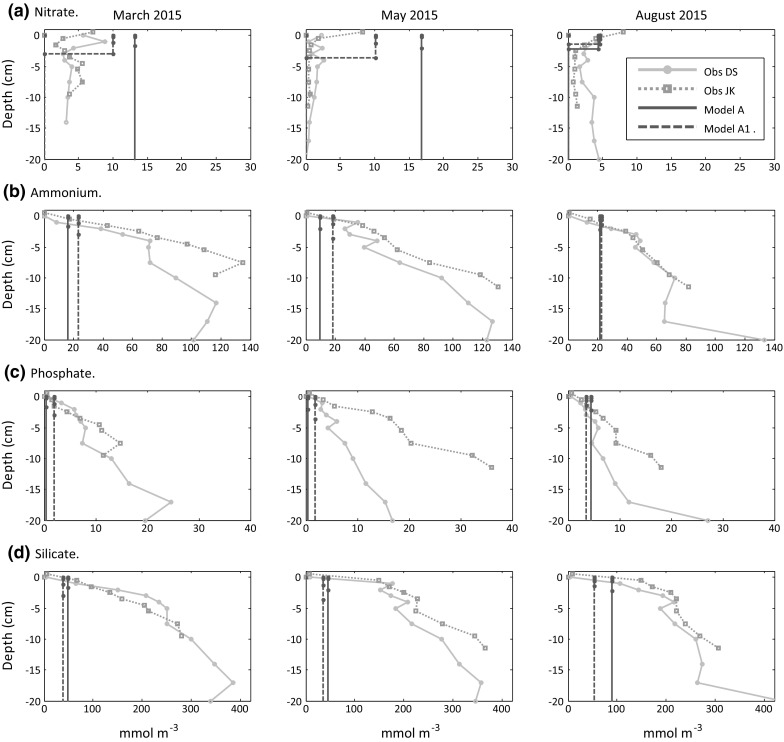
Site A, pore-water nutrient profiles (mmol m^−3^), model-data comparison. Observations, average over three replicates (*error bars* omitted for clarity). Model results are depth average concentrations. *Solid line* (Model A); *Dashed line* (Model A1). Nitrate concentrations are zero below the nitrate penetration depth (e.g. run A1 in March 2015)

Observed nitrate profiles at the muddy site A typically decreased to a minimum within the top 5 cm, but then remained constant (March 2015) or showed a slight increase with depth (August 2015). The existence of nitrate deep in the (presumably) anoxic region at site A is unexpected. There was a large variability in individual replicates, but averaged results appeared reasonably consistent between the two independent sets of measurements (DS and JK). Modelled depth average nitrate pore water concentration (Fig. 8a) at site A showed a very large overestimate for March and May 2015. The run with modified parameter settings (Model A1) gave improved agreement with near-surface concentrations and a much shallower nitrate penetration depth (25 cm Model A; 3 cm Mode A1). For both model runs, concentrations in August 2015 were comparable with observations in the near-surface layer, but observations showed presence of nitrate deeper in the cores not seen in the model. The high pore water nitrate concentrations and deep nitrogen penetration depth in Model A in March and May resulted from very low (<0.1 mmol N m^−2^ day^−1^) denitrification rates in early spring due to low anaerobic bacteria biomass as described later (see Fig. 11). When denitrification increased in August, modelled values were closer to observations. Model A1, which was parameterised to increase availability of refractory POM, had more sustained denitrification rates through the year reducing nitrate build-up in the sediment pore waters yielding results closer to observed profiles.

Measured pore water concentrations of ammonium, phosphate and silicate at site A increased with depth. In all cases, the modelled depth mean concentrations tend to be representative of observed values within the top 1–3 cm of the sediment indicating that they do not take account of the higher observed concentrations at depth, leading to substantial underestimates in depth average concentrations (model values on average 25, 15 and 30% of observed for ammonium, phosphate, and silicate respectively). This was confirmed by examining the internal layer-dependent concentrations in the model, which generally showed substantial underestimates (factor of 10) for the deeper sediment layers. It is likely that this underestimate is due to a combination of a too rapid decrease with depth of POM (Fig. 6b), too low bacterial activity, or too high sediment diffusivity. Interestingly, Model A1, which had an increased availability of POM in later winter and early spring, gave higher ammonium and phosphate (although not silicate) concentrations at this time of year compared to the reference run (Model A).

**Fig. 9:**
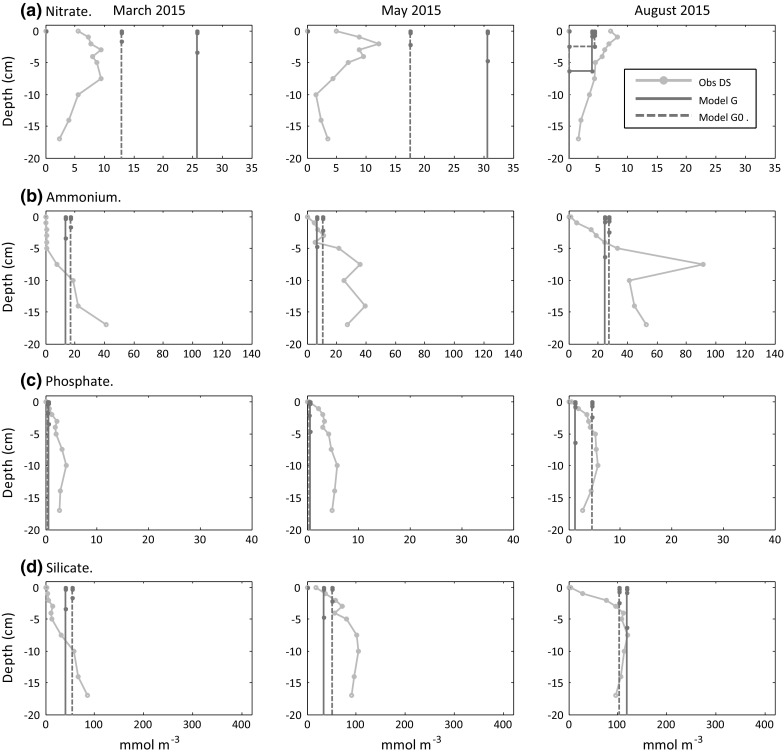
Site G, pore-water nutrient profiles (mmol m^−3^), model-data comparison. Observations, average over three replicates (*error bars* omitted for clarity). Model results are depth averaged concentrations. *Solid line* run with permeable modification (Model G); *Dashed line* no permeable sediment modification (Model G0). Nitrate, concentrations are zero below the nitrate penetration depth (e.g. run G, G0 in Aug 2015)

At the sandy site G, the observed nitrate pore water concentrations (Fig. 9a) were generally about twice that for site A, with a more gradual decline in concentration with depth compared to site A. Model nitrate concentrations in early spring were very high, again due to low denitrification rates, exacerbated by increased transport into deeper layers of the bed associated with the permeable sediment modification which increased diffusion. On a depth average basis, observed ammonium, phosphate, and silicate concentrations at G (Fig. 9b–d) were much smaller than at site A (ratio G/A, 0.4, 0.3 and 0.2 for ammonium, phosphate, and silicate respectively). Modelled pore water concentrations for these nutrients were very similar between site A and G runs, and results matched more closely with the site G measurements. For ammonium, phosphate and silicate, the permeable sediment modification had a relatively minor effect on modelled concentrations, apart from the August 2015 phosphate value, which agreed less compared with the baseline run with no modification (‘Model G0’).

### Benthic fauna and bacteria

**Fig. 10:**
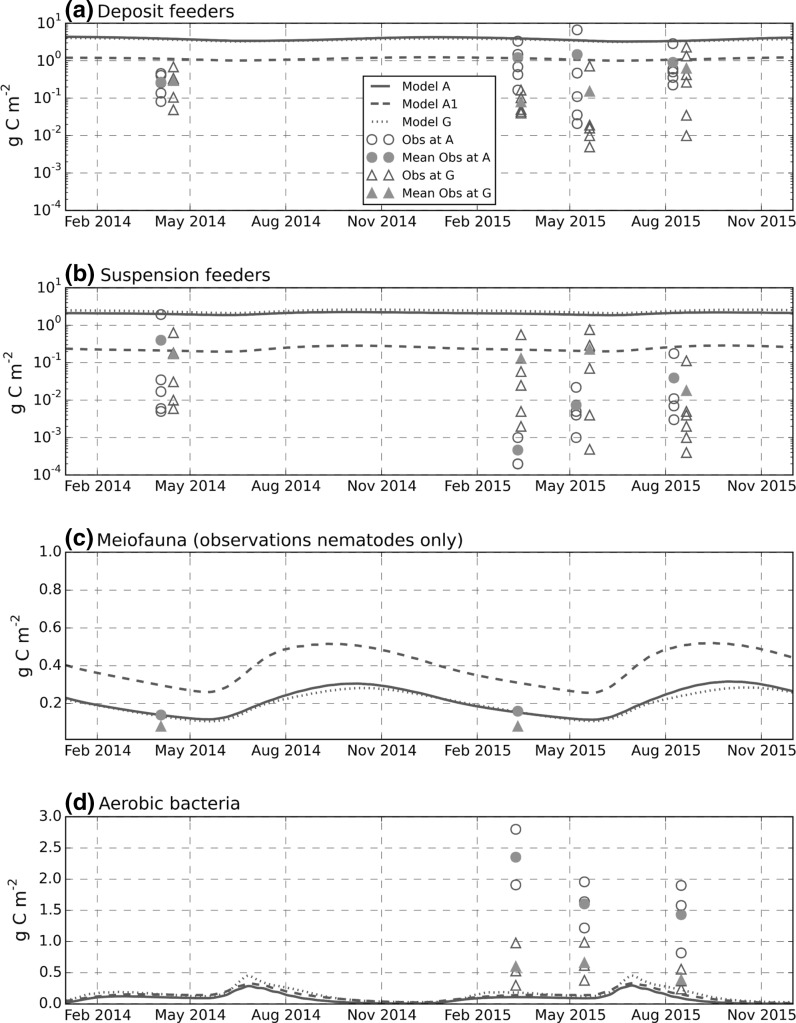
Benthic fauna and aerobic bacteria biomass (g C m^−2^). For the latter, observed values are on the top 1 cm, modelled values are over the oxygenated later depth which is variable in range 0.5–2.0 cm (Fig. 7). *Solid line* (Model A) site A with original parameter settings; *dashed line* (Model A1) site A with refractory POM modification; *dotted line* is model site G with permeable sediment formulation. Observed deposit and suspension feeder biomass shown for all replicates. Average over replicates also plotted (*solid symbols*). Meiofauna is mean value only (three replicates). Some dates offset (within a 2-week window) to avoid over-plotting of symbols

Observed macrofaunal biomass showed very large variability between replicate grab samples at the same site and time (e.g. approximate range of deposit feeder biomass: 3 × 10^−2^—8 g C m^−2^ at site A in May 2015, 1 × 10^−2^—2 g C m^−2^ at site G in August 2015; approximate range suspension feeder biomass: 8 × 10^−3^—2 g C m^−2^ at site A in April 2014, 8 × 10^−4^—0.9 g C m^−2^ at site G in May 2015). Deposit feeders generally comprised the largest biomass group in both model and observations and mean deposit feeder biomass was generally higher at site A than G. With standard parameter setting, modelled deposit and suspension-feeder biomass were at the upper end of the observed range and substantially larger than the sample mean (Fig. 10a, b). Model runs at A and G showed no significant difference. The modified run (‘Model A1’, "Model run configurations" section), with increased deposit and filter feeder mortality rates substantially improved the agreement with observations. These results are also plotted as bar charts where the relative magnitude of deposit and suspension feeders is more clearly seen (Online Resource 2).

Modelled meiofaunal biomass matched closely observed nematode biomass at the muddy site A (Fig. 10c and Online Resource 2). Observed meiofaunal biomass at G was about half that at A, while the model showed no significant difference between the sites. For the Model A1 run, meiofaunal and bacterial biomass approximately doubled due to the decreased predation from macrofauna and giving a less close agreement with the observed values.

Bacterial biomass was measured in the top 1 cm of the seabed and was therefore compared only with the aerobic bacterial component in the model (Fig. 10d). Observed bacterial biomass (in the top 1 cm) at the sandy site G was about half that of the muddy site A. The model gave very similar values for both sites. Modelled aerobic bacterial biomass was a factor of 5–10 lower than that measured at either of the sites. The run with increased macrofaunal mortality (‘Model A1’) gave no significant change to aerobic bacteria biomass.

#### Seasonal cycle and effect of modified POM breakdown

**Fig. 11:**
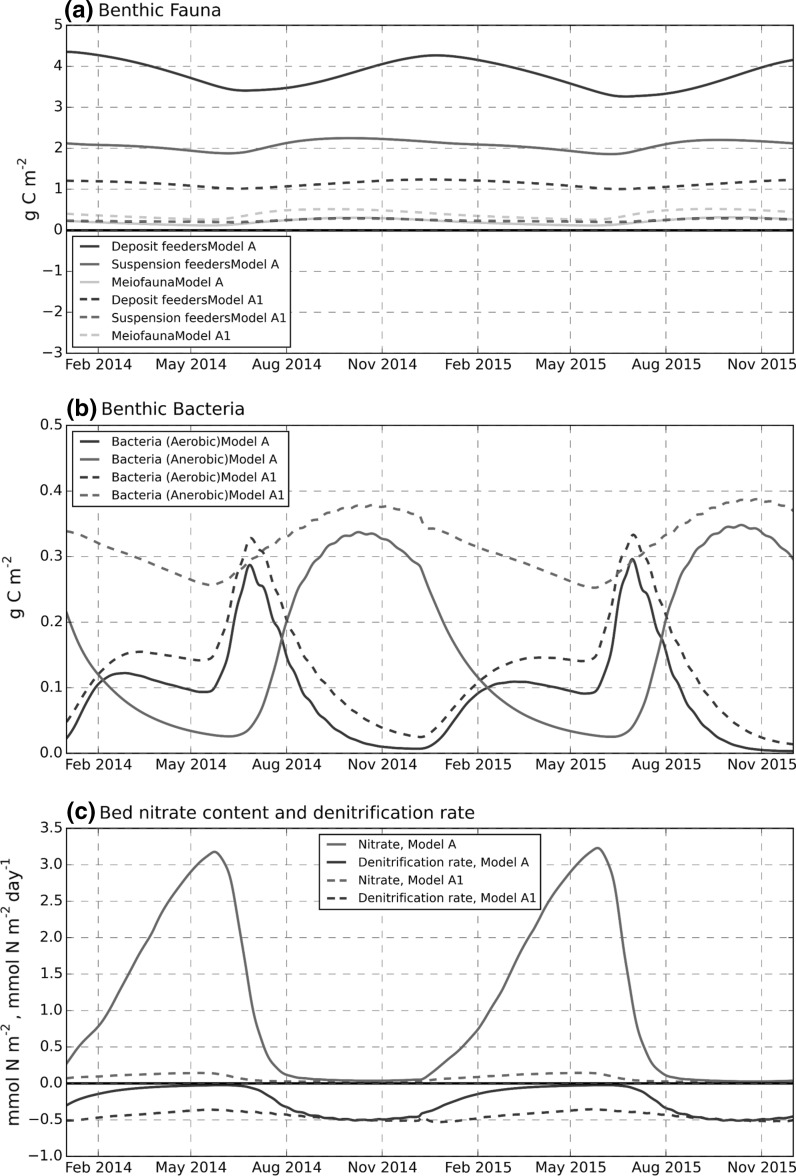
Modelled seasonal variation: **a** benthic fauna (g C m^−2^); **b** benthic bacteria (g C m^−2^); **c** bed nitrate content (mmol N m^−2^) and nitrification rate (mmol N m^−2^ day^−1^), the latter shown as a negative flux (also note mixed *vertical axis* units). *Solid line* (Model A) run with original parameter settings. *Dashed line* (Model A1)

Some further insight can be gained from time series plots of functional group biomass (Fig. 11). Aerobic bacteria biomass responded most quickly to input from the spring bloom, but then decreased rapidly through late summer, reaching low values in early winter before increasing again in January. Anaerobic bacteria showed a contrary relationship, with a minimum in late spring and peak biomass occurring in late autumn and early winter. Meiofauna and suspension feeder biomass reached a maximum in late summer and autumn, with a minimum in late spring, while peak deposit feeder biomass occurred at midwinter.

Bacterial biomass from the standard parameter run (Model A) was highly variable with > 10-fold (aerobic) and 6-fold (anaerobic) differences between minimum and maximum values over an annual cycle. The effect of allowing more bacterial consumption of refractory POM (Model A1) was to sustain anaerobic bacterial biomass and associated denitrification through winter and early spring. This had the beneficial effect of reducing the bed nitrate content closer to observed values (Fig. 8a). The aerobic bacterial biomass in Model A1 decreased, especially the peak value post spring bloom. This was due to the Model A1 parametrisation that decreased the proportion of semi-labile POM in the pelagic input. The change in POM dynamics also led to a biomass shift from suspension to deposit feeders.

In addition to the observational results shown in the main body of the paper, measured nutrient fluxes are plotted in Online Resource 3.

## Discussion

A condensed synthesis of the model data comparison based on the results and the discussion below is given in tabular form in Online Resource 4.

### Pelagic assessment

Because the benthos is driven by inputs from the pelagic system, benthic results were potentially sensitive to the modelling of pelagic physical and biogeochemical processes. Comparison with the available data indicated that physical and pelagic biogeochemistry variables most relevant for the benthic system were represented reasonably well. In particular: (1) Timing and magnitude of chlorophyll concentrations associated with the Spring bloom (Fig. 5b); (2) water column nitrate, phosphate and silicate concentrations (Fig. 3a, c, d); (3) bottom temperature (Fig. 5a). This is consistent with the generally good performance of the ERSEM model reported for pelagic variables (e.g. Blackford et al. 2004). The most notable discrepancies were (1) underestimate of surface chlorophyll concentrations post bloom (Fig. 5b), (2) a small overestimate (10%) in bottom oxygen concentrations (Fig. 5c), and (3) water column ammonium concentrations in 2014 (Fig. 3b).

Post spring bloom surface chlorophyll concentration is underestimated by the model, although for 2015, when most benthic observations were taken, agreement is closer. In the model at least, production at this time appears to be mainly at a deep chlorophyll maximum rather than the surface, so the surface underestimate may be less significant. It should also be borne in mind that surface chlorophyll concentrations are just one factor controlling supply of material to bed, other factors include sinking rates and amount of pelagic remineralisation of detrital material. Note also that chlorophyll may be a weak proxy for phytoplankton biomass (e.g. varying carbon: chlorophyll ratios; Jakobsen and Markager 2016). Crucially, benthic oxygen demand in the model is within the range of measured values (Fig. 7b) and this provides a good indication that the export of organic material to the bed is broadly correct. The overestimate in bottom oxygen concentrations (Fig. 5c) may be due to insufficient benthic oxygen uptake. However model A1, which has an uptake close to the average of the observation, did not give a significantly improved bottom oxygen prediction. This would imply that the average of the observations is an underestimate of the oxygen uptake or the model overestimate of bottom oxygen is due to something other than incorrect benthic demand. Possible causes for the latter could be a missing pelagic oxygen demand or an overestimate of oxygen flux into the bottom mixed layer through the thermocline. Some care needs to be made in interpreting results spanning the pelagic and benthic domains since the relevant oxygen concentrations were measured at the Celtic Deep 2 site, which was close to site G, but approximately 30 km from site A (Fig. 1).

Water column ammonium derives from a balance between excretion and consumption processes between bacteria, phytoplankton and zooplankton. Observations during 2014 and 2015 showed inter-annual variability in ammonium concentrations that was not captured by the model. However, model results showed reasonable agreement with observed profiles of ammonium in 2015, including evidence of production at a deep chlorophyll maximum, with the main discrepancy being the increase in bottom concentrations between May and July 2015 compared with an observed decrease (Fig. 4). The latter result is consistent with measurements at site A showing an ammonium flux *into* the bed in August 2015 (Kitidis et al. 2017), although this is not consistent with observed ammonium pore water profiles (Fig. 8b), which, in accord with the model, suggest a flux out of the bed at this time. The increase in modelled bottom ammonium concentrations includes contributions from the benthic system, (possible) production within the bottom mixed layer itself and production at the top of the layer near the deep chlorophyll maximum. A model sensitivity run to see if the ammonium flux out of the bed was the primary cause of ammonium increase was inconclusive, as it was not possible to independently isolate and control a single flux in a coupled model without affecting the other coupled fluxes and state variables.

### Organic matter

An interesting result is the difference between observed quantities of organic material in the seabed and the equivalent in the model (Fig. 6). This suggests that either a large part of the observed organic carbon in the bed is biologically inactive, or there is biogeochemical activity taking place that is not properly represented in the model. To explore this further, a simple mass balance model is considered. If *Q* (g m^−2^) is the quantity of POM down to a fixed depth of sediment with an annual cycle of input from the water column *p*(*t*) (g m^−2^ y^−1^) (arising ultimately from primary production), and if the remineralisation rate of *Q* into inorganic forms is *l*(*t*) (g m^−2^ year^−1^), then, by definition, the rate of change of POM is:

7 $$dQ/dt = \, p{-}l$$

Assuming this is a linear equation, then if p(t) is an (approximately) annually repeating function, Q will be as well. Then integrating over an annual cycle, the annual change in Q is given by ΔQ = P − L, where P = ∫*p*(*t*)*dt* and L = ∫*l*(*t*)*dt*. If the bed is near equilibrium, i.e. ΔQ is small compared to P, then L ~ P so that near equilibrium, the benthic system reaches a state where consumption of organic material matches the input. Annual rates related to consumption of POM (i.e. nearly all oxygen consumption rates, nutrient fluxes, faunal growth) are thus set by the input rate P, not the ‘standing stock’ Q. States such as concentrations and biomass, are ultimately determined by rates (plus physical constants and boundary conditions). This suggests that it is possible to reproduce overall magnitudes of observed fluxes and states if the modelled POM input is approximately correct, independent of the model standing stock of benthic POM.

Further insight is gained, if it is assumed that the flux L can be approximated as a 1st order process proportional to the amount of benthic POM. If L = λ Q, where λ (units y^−1^) is some measure of the timescale for the conversion of organic material to inorganic forms, then the same magnitude of L can be achieved by a small standing stock being broken down relatively rapidly (small Q, large λ) or a large standing stock being consumed slowly (large Q, small λ). Roughly, this corresponds to the two alternatives outlined above: either (1) much of the observed organic material in the bed being inactive, with the modelled stock being representative of the biologically active component (small Q, large λ), or (2) most of the benthic POM is active, albeit with a very slow degradation rate (large Q, small λ). For the latter case, the implication would be that rates of consumption of POM in the model are too high, leading to a depleted POM stock in the bed. However, it is argued that the former is more likely to be the case. The rates of biological consumption used in the model (Ebenhoh et al. 1995; Blackford 1997) include macro faunal growth rates based on scaling laws related to assumed body sizes for each functional group (Fenchel 1974). Also, bacterial processes were set by reference to experimental observations that, although subject to uncertainty, are unlikely to be wrong by the magnitude required to explain the difference between the observed and modelled bed POM content.

Note, the arguments in terms of annual averages discussed above do not apply for behaviour at shorter timescales. Very slow degradation rates (small *λ*) would lead to a highly damped response to inputs and little seasonal variation in fluxes from POM decomposition. Conversely, rapid degradation rates would give rise to a strong seasonal variation in *l*(*t*), reflecting the seasonality of the input *p*(*t*), for example high oxygen uptake or fluxes of inorganic nutrients after the spring bloom. In principle, this could be seen in the observations. In this regard the low temporal resolution of the present dataset (3 measurement over a year), together with high variability between replicates is not ideal for picking out seasonal signals. However, observed oxygen consumption (Fig. 7b) generally showed an increase during and after the spring bloom consistent with a fast rather than highly damped response to pelagic inputs.

Of interest is the (close to Redfield) molar C: N ratio (~6.6) in the model at both sites (Fig. 6a), that would be appropriate for very labile OM. Observations of C: N ratios at site A are more N depleted (C: N ~ 11). This compares to between 9 and 10 at muddy sites in the North Sea (Defra 2013), and suggests possibly older, refractory material at the North Sea sites. Site A potentially has a large historic pool of carbon which is what was measured, whereas the model seems to be driven largely by recent phyto-detrital carbon.

If it is the case that much of the observed POM is inert, then if the model profiles are approximately correct for the non-inert portion, Fig. 6b suggests that even in the top 1–2 cm the majority of the POM at the muddy site A might be relatively inert. This could be a consequence either of fresh inputs containing large quantities of highly refractory material, and/or, strong mixing of the top seabed layer with older material deeper in the bed.

An order of magnitude calculation is helpful to assess the feasibility that a large quantity of measured organic carbon in the bed is biologically inert. Assuming net primary productivity (PP) in temperate shelf seas of 1000–200 g C m^−2^ year^−1^ with ~20 g m^−2^ y^−1^ entering the benthic system (Joint et al. 2001) then if 1 g m^−2^ year^−1^ (5% of benthic input, 0.5–1.0% of net PP) becomes deeply buried (or otherwise too refractory for biological breakdown) then values of ~2400 g C m^−2^ observed at site A could be achieved within 2000–3000 years, which is not unreasonable. In terms of the ERSEM 15.06 model, this biologically inert material could be identified with the buried material component (Fig. 2a) and, since this plays no role in the model dynamics, the model could be trivially fitted to observed POM values by setting an appropriate initial value. Evidence on the rate at which this inactive fraction is produced, and controlling mechanisms in relation to shelf sea conditions and location, will constitute important information to quantify changes in carbon cycling and ultimately sequestration.

### Oxygen

Modelled benthic oxygen uptake was within, but toward the lower end, of the observed range of values (Fig. 7b) while oxygen-penetration depth was overestimated by a factor of 2 in spring and early summer at the muddy site A (Fig. 7c). The relatively small (10%) overestimate of near-bed oxygen in the model (Fig. 5c) would be expected to contribute only a proportionate amount to the overestimate in OPD. This was confirmed by a model sensitivity run (not shown) where bottom oxygen matched the observed values, but yielded only a very small improvement in OPD. This result suggests that differences in OPD were mainly due to underestimates of benthic oxygen consumption. The alternative parameterisation (Model A1) yielded a more even supply of degradable POM through the year, improving the agreement with TOU and OPD in late winter and early spring. The effect of the permeable sediment modification on the oxygen dynamics is discussed later.

### Pore water nutrients

The model nitrate concentrations within the top 1–2 cm were comparable to observed values in August 2015, but were greatly overestimated (factors of 10 and 3 at A and G respectively) at all depths in March and May 2015 (Figs. 8a, 9a) due to low rates of nitrate removal via denitrification; this in turn was related to decreased aerobic bacteria biomass. The modified run (Model A1), with increased breakdown of benthic POM in winter and spring, maintained anaerobic bacterial biomass and denitrification, eliminating the very deep nitrate penetration depth, and yielding nitrate pore water concentrations more comparable with those observed, although still overestimated in the near-surface layer. Measurements (Kitidis et al. 2017) indicate that anammox rather than denitrification dominates nitrogen removal at site A, while at site G rates for both denitrification and anammox processes were very low. The absence of the annamox pathway in the model makes detailed comparison of model results with nitrate and ammonium pore water concentrations problematical. Nevertheless, the comparison between model runs ‘A’ and ‘A1’ (Fig. 11) highlights a general point about the relationship between nitrification, denitrification, bacteria, and organic matter in the model. Very large nitrate concentrations can arise in winter and early spring by a combination of low denitrification rates due to reduced anaerobic bacteria biomass caused by a rundown over winter of the available POM pool. Model sensitivity runs (not shown) indicated that reductions in the within-bed diffusivity could also help reduce model nitrate concentrations in the winter/early spring period.

Observed pore water concentrations for ammonium, phosphate and silicate at site A increased strongly with depth in the sea bed. At this site, the modelled depth average concentration was generally comparable with observed values near the sediment surface but was substantially smaller than obsereved concentrations deeper in the sediment (Fig. 8a–c). Observed concentrations at the sandy site G were less than at A and model results were closer to observations here (Fig. 9a–c). The underestimate in modelled concentrations deeper in the sediment could be the result of a number of factors: (1) too rapid decrease in model OM with depth (Fig. 6b), (2) breakdown of POM by anaerobic bacteria that is too low, and/or (3) within-bed diffusion that is too high. The observed increase with depth of pore water concentrations for ammonium, phosphate and silicate indicates that degradation of OM is occurring down to the core depth of 25 cm at site A. This does not accord with a conceptual picture of a relatively shallow active layer of OM breakdown with highly refractory material buried below, but in conjunction with the discussion on POM above, suggests a more homogenous 20–30 cm layer extending from the surface comprised of a mixture of both highly refractory and semi-labile material. This contrasts with site G, where ammonium, phosphate and silicate profiles showed a decrease in concentration below around 10 cm, suggesting a shallow layer of degradable organic material.

### Fauna and bacteria

With the initial parameter settings, the model macrofaunal biomass was significantly higher than observed. Observed biomass (combined infaunal and epifaunal macrofaunal values of 38.0 and 17.8 wet weight g m^−2^ for site A and G respectively, Thompson et al. 2017), appear to be at the low end of what is observed more generally on the European Continental Shelf. Bolam et al. (2010) found an average benthic wet weight biomass of 61 (±11) g m^−2^ based on 155 sediment cores sampled in the southern North Sea, English Channel, Celtic Sea, Irish Sea, and Malin Shelf. Although site A is known to be heavily trawled (Thompson et al. 2017), trawling disturbance seemed unlikely as an explanation for the relatively low biomass since biomass was uniformly low at all sites in the study region and broad measures, such as average biomass, appear to show little correlation with trawling-intensity estimates (Thompson et al. 2017). The average overestimate in modelled macrofaunal biomass in model A and G although large (factor of 10) (Fig. 10a, Online Resource 2) is at the limits seen in previous studies (Ebenhoh et al. 1995). The run with increased mortality rates for deposit and filter feeders, yielded carbon biomass values for these groups that were closer to those observed (Fig. 10). Interestingly this had almost no effect on oxygen consumption (which remained at a similar magnitude to that observed) due to a compensating increase in meiofaunal biomass. It is hypothesised, that modelled oxygen uptake is rather insensitive to the relative biomasses of the biological components (macrofauna, meiofauna and bacteria), but is ultimately controlled by total input of organic material to the bed.

Meiofaunal biomass in the model was almost exactly the same as the average value measured in late winter in 2014 and 2015 at site A and approximately double that measured at G. In contrast, modelled aerobic bacterial biomass (0.1–0.3 g C m^−2^) was much lower than the observed values (0.5–2.5 g C m^−2^) in the top 1 cm of the bed (Fig. 10b). However, the method used to estimate bacterial biomass can include dormant bacteria, while the model value is associated with active bacteria. An underestimate in modelled bacterial biomass is also reported in Blackford (1997) in the North Sea. The run with increased macrofaunal mortality (Model A1) led to an increase in meiofaunal biomass that worsens the agreement with observations, although there were only two measurements of this quantity, both in later winter/early spring. A limited set of further sensitivity runs were not successful in significantly increasing the model aerobic bacterial biomass.

Taken at face value, these results suggest that for modelling these sites, a rebalancing of benthic biomass from larger to smaller organisms may be desirable. Although site specific comparisons are useful, any general recalibration of the faunal parameters needs to be based on a spatially extensive dataset to avoid biasing. For example, Blackford (1997) used the North Sea Benthos Survey to compare spatial distribution of macrofaunal biomass with 3D ERSEM predictions. The recent data set presented by Bolam et al. (2010), that takes account of benthic productivity as well as biomass, could form a basis for this task. More generally, the risk of over-calibrating from a limited set of locations applies to all model variables. For this, recent spatial data for OPD (Defra 2013) and benthic carbon (Diesing et al. 2017) could form a key resource for model improvement and validation.

### Permeable sediments

The simple approach used to include permeable sediment effects within the framework of the current ERSEM 15.06 model met with mixed success. The additional term acting as a proxy for the effect of pore water flow increased diffusivity by between 40% (Neap tides) to 70% (Spring tides). The modification worked best in reproducing the deeper oxic layers associated with site G observations but required calibrating the scaling constant in Eq. 2. With this value, the model matched OPD magnitudes at the sandy site G in 2015 reasonably well (Fig. 7c), reproducing the observed changes in depth (5 cm in March 2015–1 cm in August 2015). However, the detailed timing behaviour was poor leading to an underestimate in March (5 cm observed, 3 cm modelled) and a significant overestimate in May 2015 (1 cm observed, 5 cm modelled). Observed OPD changed markedly at this site between years (2 cm in March–April 2014, and 5 cm in March 2015) which could not be captured by the model.

The permeable-sediment modification to diffusivity had a limited effect on total oxygen demand, principally a small (<10%) enhancement immediately after the spring bloom (Fig. 7b) caused by increased aerobic bacteria consumption arising from a combination of a deeper oxic layer, allowing aerobic bacteria access to greater depth of organic material, and increased benthic inputs of labile and semi-labile organic matter at this time. The deepening of the oxic layer alone was not sufficient, as indicated by the deeper layer prior to the spring bloom with no obvious increase in oxygen consumption. Enhanced oxygen uptake in permeable sediments has been observed in several studies (Janssen et al. 2005; Cook et al. 2007), although observations presented here (data ‘NH’ Fig. 7b; also Hicks et al. 2017) found lower values for TOU at the sandy site G compared with site A. However, measurements at G were not conducted under conditions simulating pore water flows and may underestimate the oxygen demand generated by oxic respiration (Polerecky et al. 2005).

A possible mechanism contributing to observed increases in benthic oxygen uptake not implemented in the model, is the drawing in of phytoplankton, DOC and fine POC from the benthic boundary layer by advective pore water exchange (Ehrenhauss et al. 2004; Chipman et al. 2010). Implementation in the model may increase oxygen uptake and associated remineralisation of organic material. However, as discussed under "Fauna and Bacteria" section above, if annual oxygen consumption is ultimately controlled by total water column production, this may only lead to a temporary increase in benthic oxygen demand. An overall increase will only occur only if more rapid benthic return, e.g. of nutrients, leads to an increase in annual water column production.

## Summary and conclusions

The site-specific nature of the comparison means care must be taken in drawing too general a set of conclusions. Nevertheless, the main findings of the study are summarised here.

1. The 1D GOTM-ERSEM water column model, with some site-specific adjustments, generally represented observations of pelagic variables well and provided good support for the benthic model.
2. Total oxygen uptake in the model was within the observed range at both the muddy and sandy sites, although the oxic layer depth is overestimated before and during the spring bloom at the muddy site. Changes to OM bacterial breakdown rates at site A improved agreement with measured values.
3. Total oxygen uptake appeared insensitive to the relative proportion of macrofauna, meiofauna and bacteria biomass in the model, with changes in one functional group being compensated by another to maintain an oxygen demand that, it is suggested, is ultimately determined by the rate of organic matter input.
4. The active benthic organic matter pool in the model is essentially new material from the last spring–summer pelagic input and is sufficient to support levels of TOU and biomass of the order of those observed. However, observed quantities of benthic organic carbon were up to two orders of magnitude greater than this active model pool. It is suggested that much of this observed carbon material is old and being broken down slowly or not at all. Evidence on the rate at which this inactive fraction is produced will constitute important information to quantify carbon sequestration in shelf seas.
5. Modelled pore water nitrate concentration in winter and spring became extremely high compared with observations. This was because of reduced nitrate removal to N_2_ and occurred when bacterial biomass became small due to reduced benthic POM availability prior to the spring bloom.
6. Modelled depth average pore water concentrations of ammonium, phosphate and silicate at the muddy site A were 5–50% of observed values due to an underestimate of concentrations associated with the deeper sediment layers. At the sandy site G, observed pore water concentrations of ammonium, phosphate, and silicate decreased below around 10 cm and were generally closer to modelled values (model values 15–150% of observed values). Observations at site A showed increasing concentrations of these nutrients to the depth of the core samples (25 cm) indicating that nutrient production is occurring at this depth in the sediment. In conjunction with conclusion 4, this suggests that at this site, relatively labile as well as highly refractory material is present even relatively deep in the sediment.
7. Modelled macrofaunal biomass was overestimated at both sites by factors in the range 3–10. Although modifications to macrofaunal mortality rates gave total macrofaunal biomass comparable to observed values at site A, it is suggested that use of large scale spatial datasets rather than ad-hoc adjustments at a single site is the way forward. Comparison with measured bacterial biomass suggested model values were too low, but the conclusion was tentative due to the difficulty of distinguishing, in the observations, between active and dormant bacterial biomass.
8. The permeable sediment modification led to an increase in oxic layer depth similar to those observed at the sand site in 2015, and a small short term increase in oxygen uptake rate. However, it did not lead to improved agreement with observed pore water nutrient and faunal biomass at the sandy site. Future work should consider pore water exchange of dissolved and fine particulate material into the bed as a possible mechanism to reproduce observed increases in oxygen uptake in permeable sediments.
9. The modelled benthic biogeochemistry showed substantial seasonal variability that was difficult to verify with the low temporal resolution of the observations and often high variability between replicates. Future observations with higher temporal frequency would be recommended to best advance understanding and aid model development.
10. Given the observed occurrence of significant anammox processes, inclusion of these processes should be considered in benthic biogeochemical models of shelf seas.

It is suggested that future developments in ERSEM should include: revisiting the parameterisation of the breakdown and mixing of OM in the bed; validation of faunal biomass based on observations over a large spatial area; and consideration of incorporating the anammox pathway in the nitrogen cycle.

## Electronic supplementary material

Below is the link to the electronic supplementary material.

Supplementary material 1 (PDF 656 kb)

Supplementary material 2 (PDF 386 kb)

Supplementary material 3 (PDF 369 kb)

Supplementary material 4 (PDF 289 kb)

## Footnotes

1 Soulsby (1997) Eq. 90, *z*_*0*_ = *a*_*r*_ *h* ^*2*^ */L* with ripple slope estimate h/L ~ 1/7 and a_r_ = 1.0. The grain roughness contribution to z_0_ was neglected on the assumption it was small compared to the bedform contribution.
