# Supplementary material for: Comparing benthic biogeochemistry at a sandy and a muddy site in the Celtic Sea using a model and observations
Source: Biogeochemistry. 2017 Sep 7;135(1):155–82. doi: 10.1007/s10533-017-0367-0 (PMC6961523; doi:10.1007/s10533-017-0367-0)
Supplement: Supplementary file 1 — Supplementary material 1 (PDF 656 kb) [file 10533_2017_367_MOESM1_ESM.pdf]

Comparing benthic biogeochemistry at a sandy and a muddy site in the Celtic Sea using a model and observations

Aldridge J.N.* [1], Lessin G.[2], Amoudry L. O.[5], Hicks N.[3], Hull T.[1], Klar J.[4], Kitidis V.[2], McNeill C.L.[2], Ingels J.[6], Parker R[1], Silburn B[1]., Silva T.[1], Sivyer D. B.[1], Smith H.[4], Widdicombe S.[2], Woodward E.M.S.[2], van der Molen J.[1], Garcia L.[1], Kroeger S.[1]

Online Resource 1: Set of depth-time figures for modelled pelagic variables.

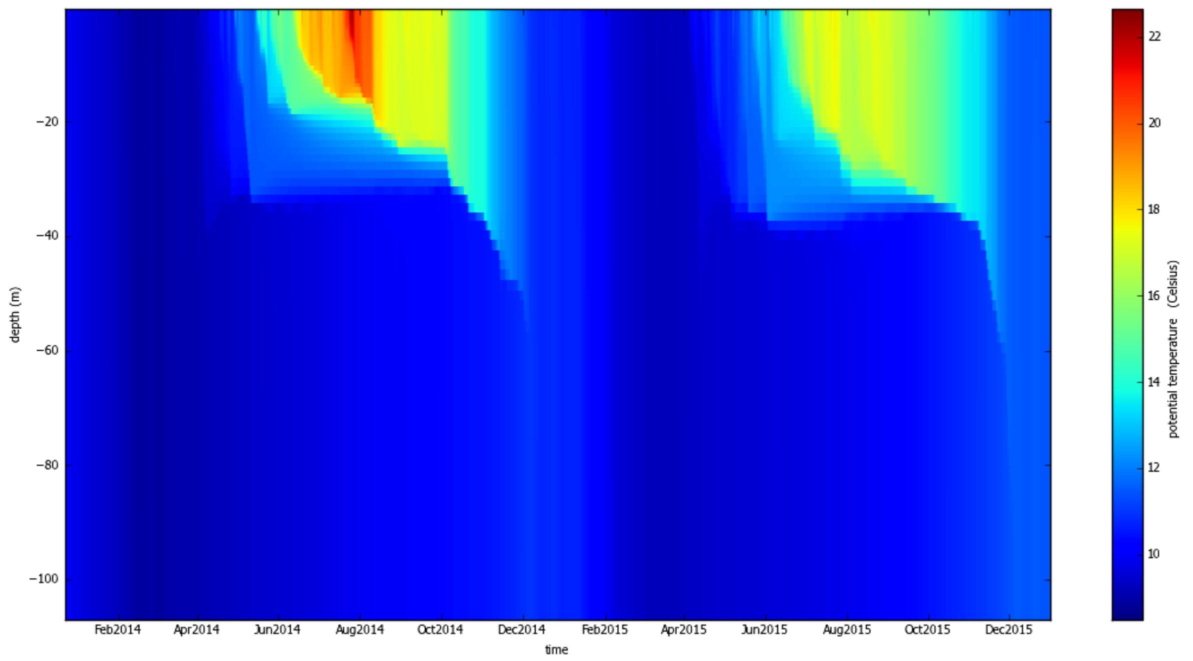

Figure A: Model temperature (° C) against depth for 2014-2015, run at site A with original benthic parameter settings (model A). Results show onset of stratification and deepening of the thermocline through the year until water column remixes. Significant inter-annual variability is evident between 2014 and 2015 with 2015 temperatures in the upper mixed layer significantly cooler in 2015.

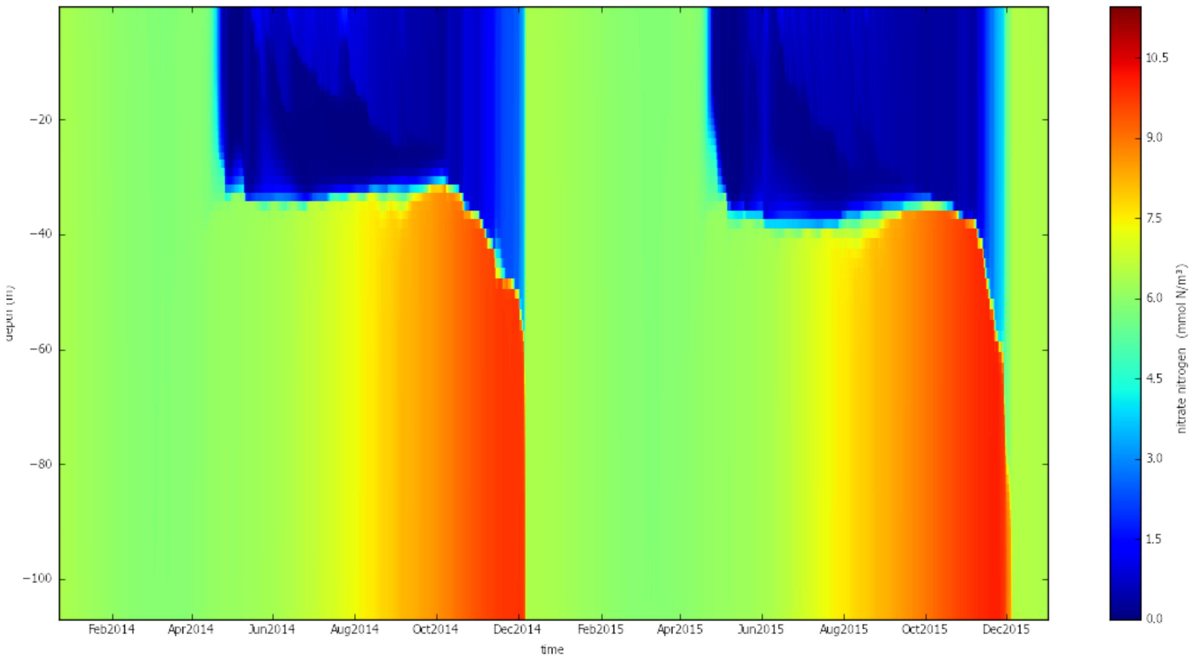

Figure B: Nitrate (mmol m^{-3}) against depth for 2014-2015, run at site A with original benthic parameter settings (model A). Results show onset of stratification and consumption of nitrate in the upper mixed layer. In the bottom mixed layer nitrate concentrations increase over the period of stratification due to remineralisation of organic material in the water column and the seabed. Little inter-annual variability is evident between 2014 and 2015.

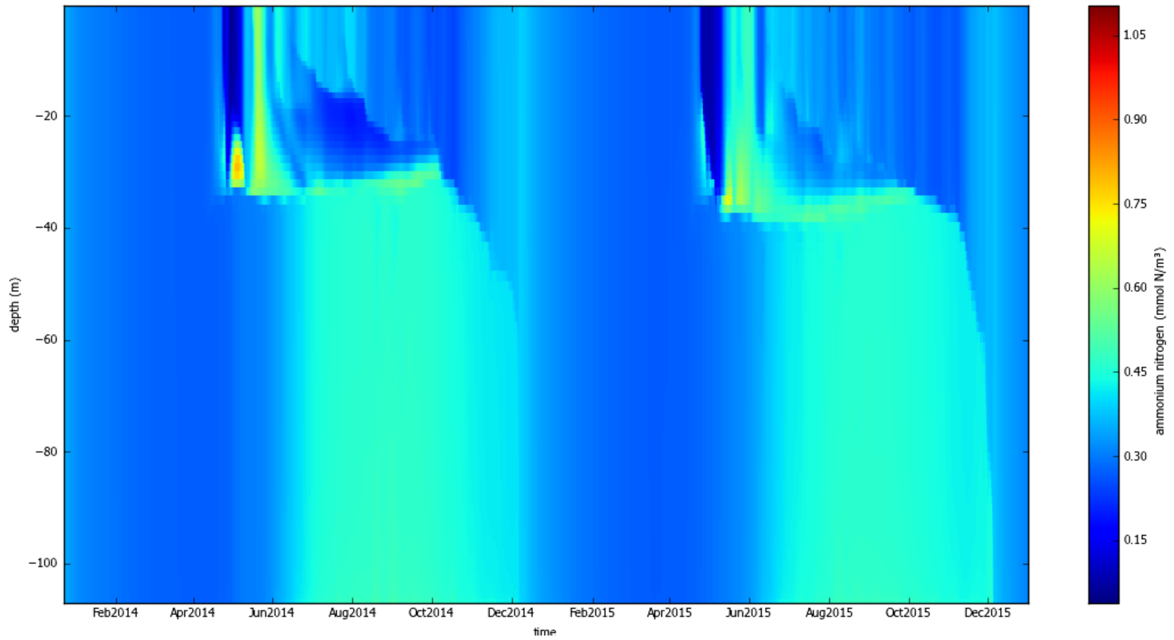

Figure C: Ammonium (mmol m^{-3}) against depth for 2014-2015, run at site A with original benthic parameter settings (model A). Results show onset of stratification and complex pattern of consumption and production of ammonium in the upper mixed layer. In the bottom mixed layer ammonium concentrations increase over the period of stratification due to remineralisation of organic material in the water column and the seabed. Some inter-annual variability in the upper mixed layer is evident between 2014 and 2015.

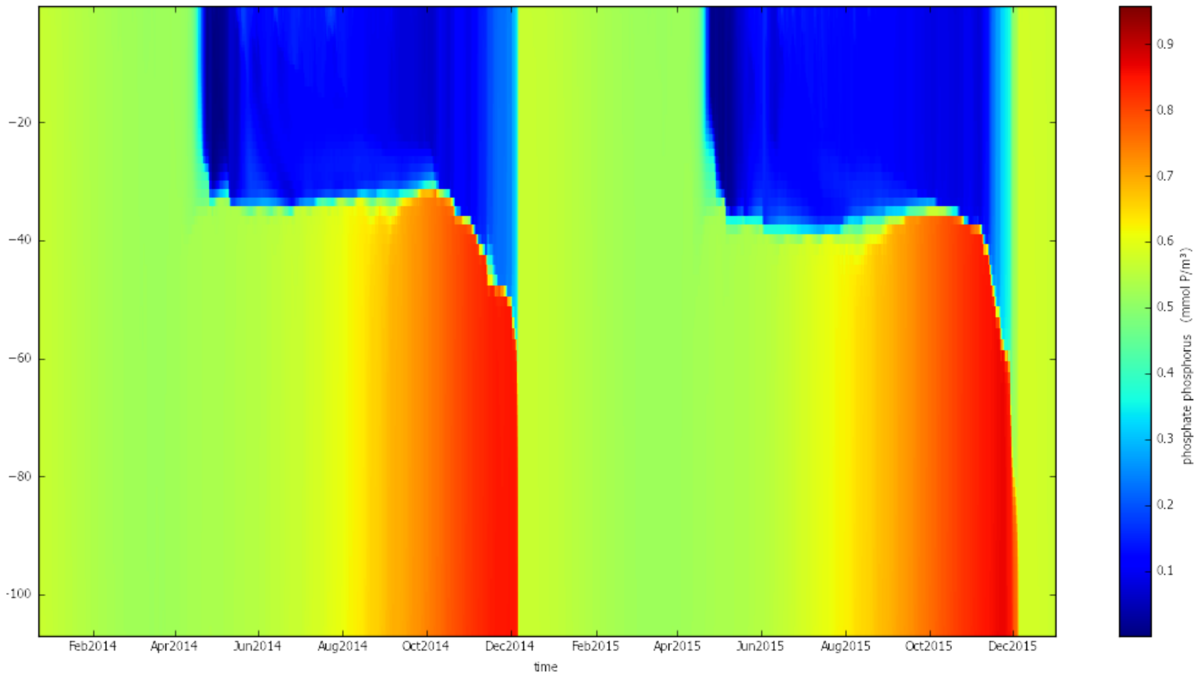

Figure D: Phosphate (mmol m^{-3}) against depth for 2014-2015, run at site A with original benthic parameter settings (model A). Results show onset of stratification and consumption of phosphate in the upper mixed layer. In the bottom mixed layer phosphrate concentrations increase over the period of stratification due to remineralisation of organic material in the water column and the seabed. Little inter-annual variability is evident between 2014 and 2015.

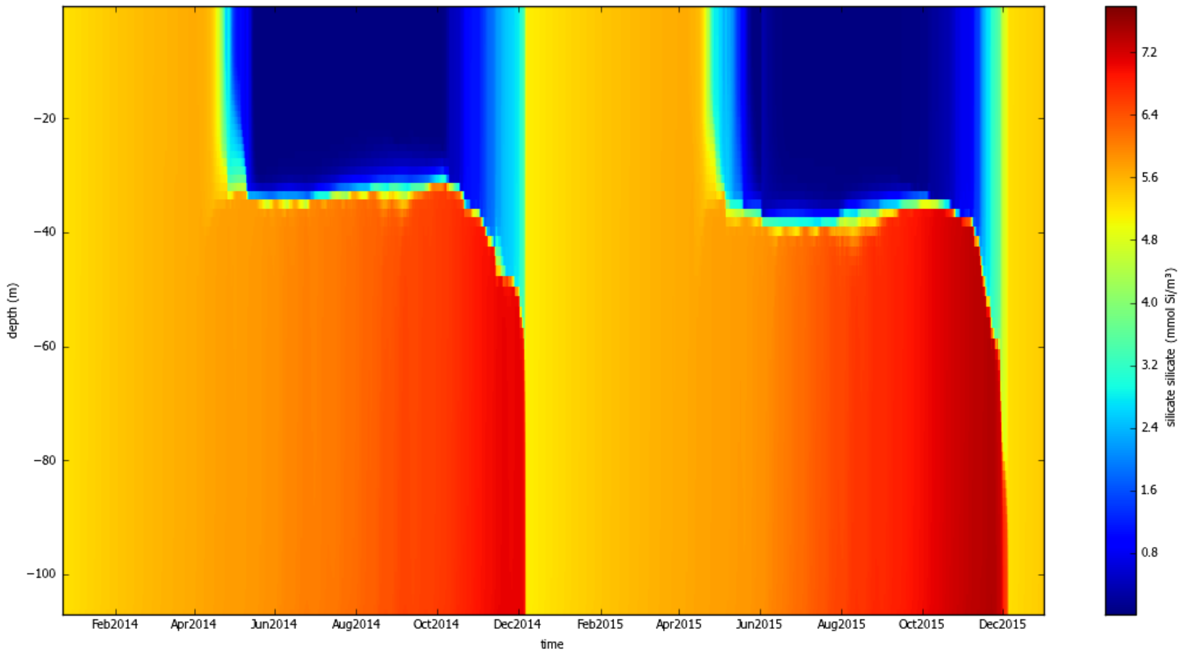

Figure E: Silicate (mmol m^{-3}) against depth for 2014-2015, run at site A with original benthic parameter settings (model A). Results show onset of stratification and consumption of silicate in the upper mixed layer. In the bottom mixed layer silicate concentrations increase over the period of stratification due to remineralisation of organic material in the water column and the seabed. Little inter-annual variability is evident between 2014 and 2015.

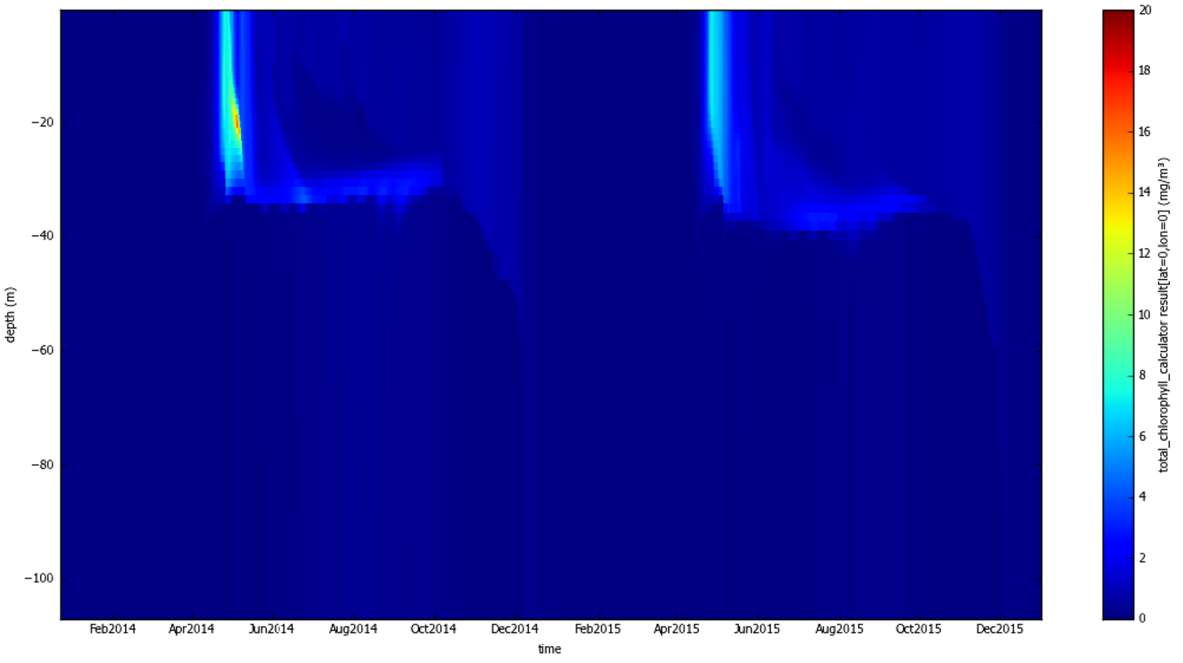

Figure F: Chlorophyll (mg m^{-3}) against depth for 2014-2015, run at site A with original benthic parameter settings (model A). Results show the spring bloom after which highest concentrations occur at the base of the upper mixed layer (deep chlorophyll maximum). Some inter-annual variability is evident between 2014 and 2015 with indications of a stronger spring bloom in 2014.

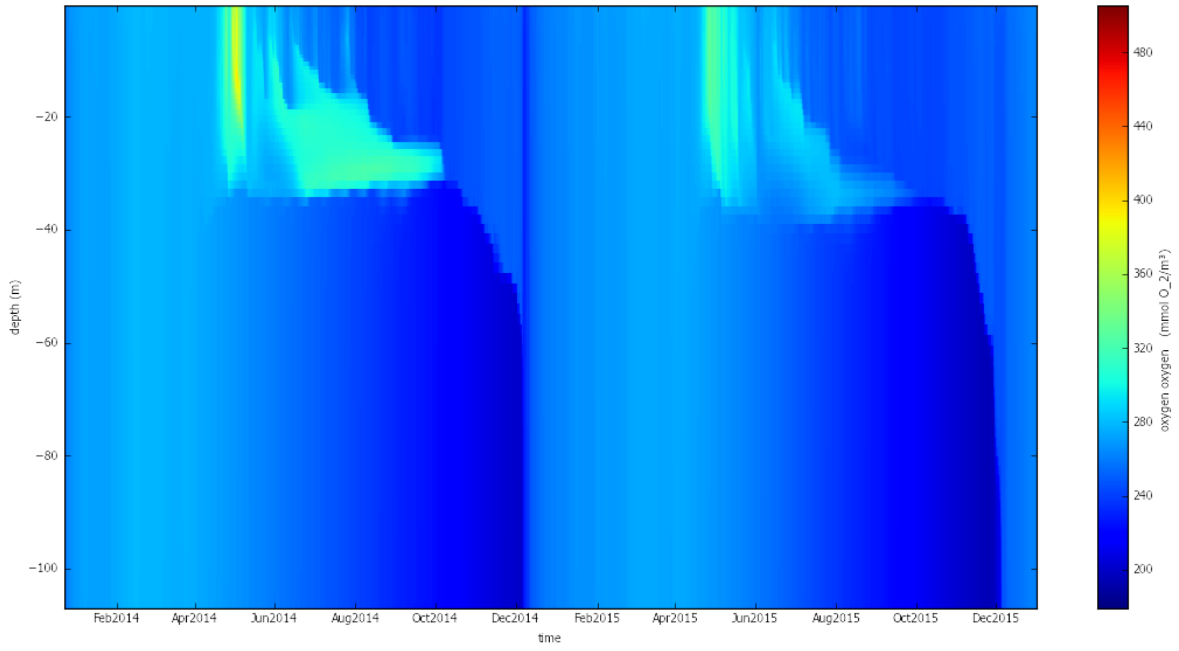

Figure G: Oxygen (mmol m^{-3}) against depth for 2014-2015, run at site A with original benthic parameter settings (model A). Results show enhanced oxygen associated with the spring bloom and at the deep chlorophyll maximum. Some inter-annual variability is evident between 2014 and 2015 with indications of a stronger oxygen production in 2014.
