# Supplementary material for: Comparing benthic biogeochemistry at a sandy and a muddy site in the Celtic Sea using a model and observations
Source: Biogeochemistry. 2017 Sep 7;135(1):155–82. doi: 10.1007/s10533-017-0367-0 (PMC6961523; doi:10.1007/s10533-017-0367-0)
Supplement: Supplementary file 2 — Supplementary material 2 (PDF 386 kb) [file 10533_2017_367_MOESM2_ESM.pdf]

Comparing benthic biogeochemistry at a sandy and a muddy site in the Celtic Sea using a model and observations

Aldridge J.N.* [1], Lessin G.[2], Amoudry L. O.[5], Hicks N.[3], Hull T.[1], Klar J.[4], Kitidis V.[2], McNeill C.L.[2], Ingels J.[6], Parker R[1], Silburn B[1]., Silva T.[1], Sivyer D. B.[1], Smith H.[4], Widdicombe S.[2], Woodward E.M.S.[2], van der Molen J.[1], Garcia L.[1], Kroeger S.[1]

Online Resource 2: Alternative plots of faunal and bacterial data and model comparison.

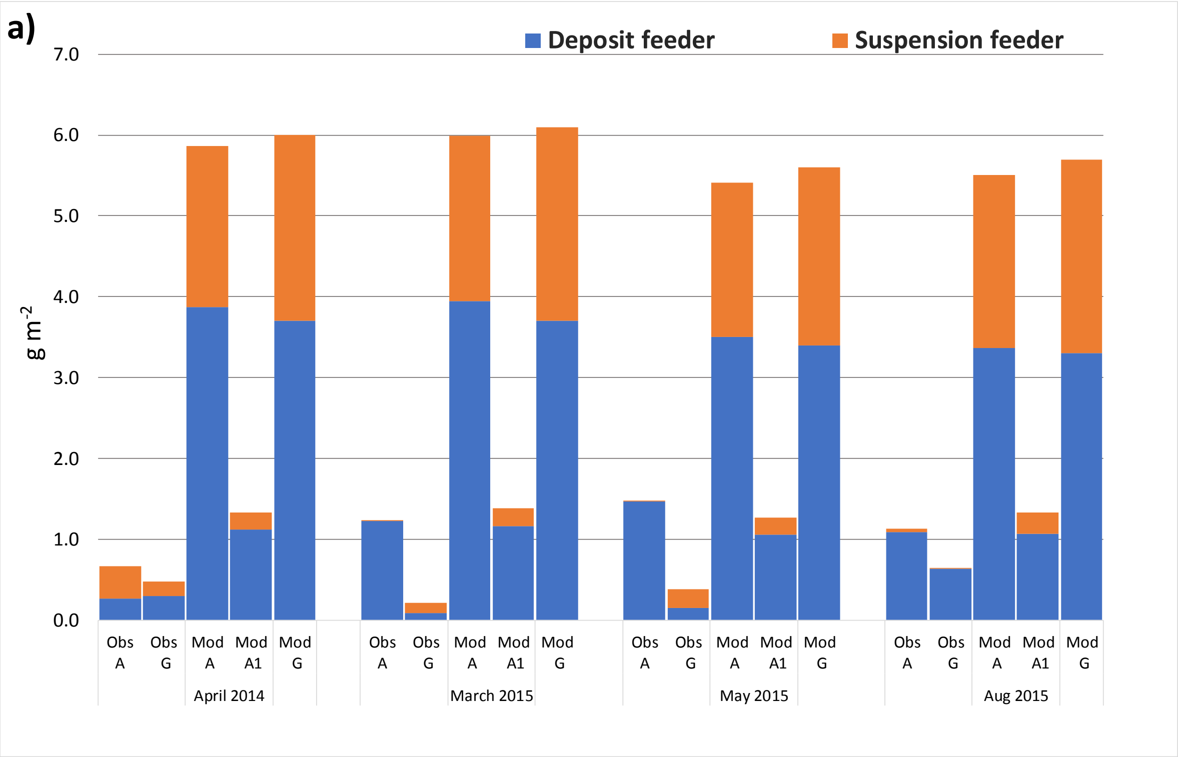

Figure a: Benthic macrofauna model-data comparison at sites A and G. Observations replicate means (5 samples).

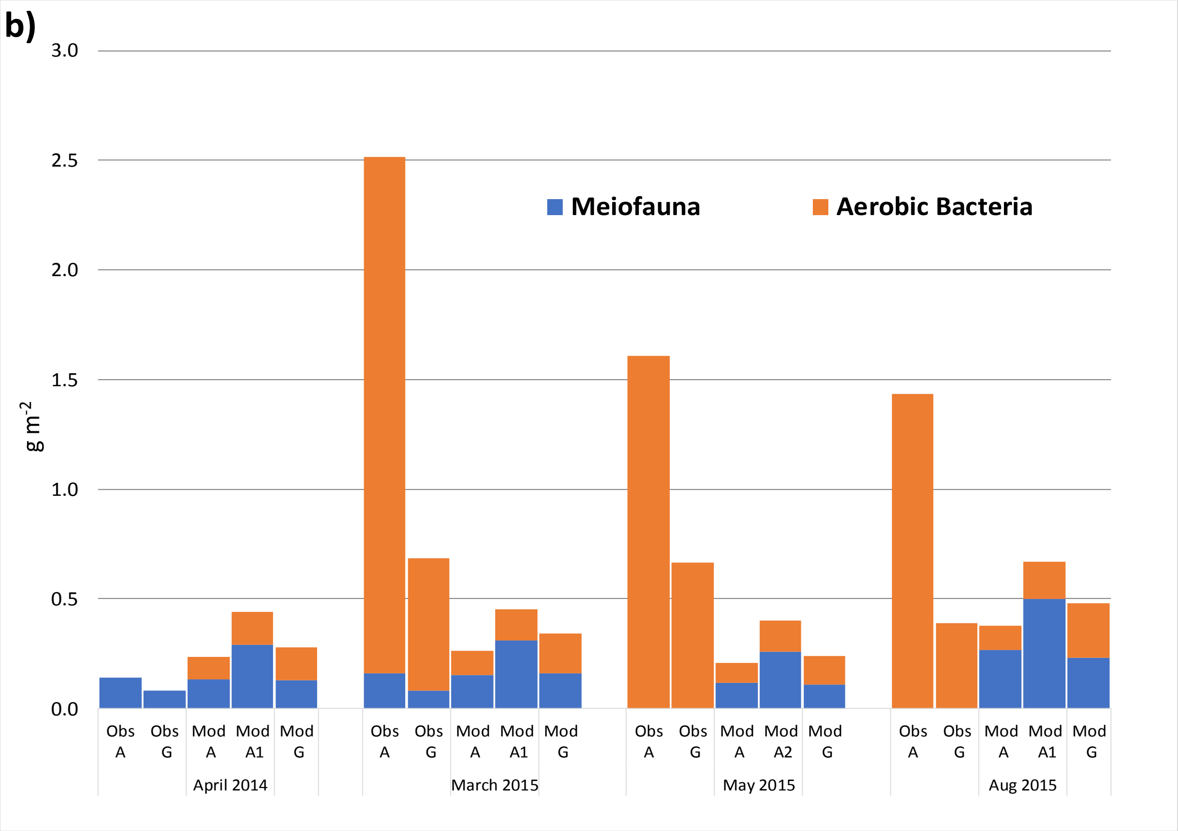

Figure b: Meiofaunal and aerobic bacteria biomass (g C m^{-2}) model-data comparison at sites A and G. Observations replicate means (5 samples).
