# Supplementary material for: Comparing benthic biogeochemistry at a sandy and a muddy site in the Celtic Sea using a model and observations
Source: Biogeochemistry. 2017 Sep 7;135(1):155–82. doi: 10.1007/s10533-017-0367-0 (PMC6961523; doi:10.1007/s10533-017-0367-0)
Supplement: Supplementary file 3 — Supplementary material 3 (PDF 369 kb) [file 10533_2017_367_MOESM3_ESM.pdf]

Comparing benthic biogeochemistry at a sandy and a muddy site in the Celtic Sea using a model and observations

Aldridge J.N.* [1], Lessin G.[2], Amoudry L. O.[5], Hicks N.[3], Hull T.[1], Klar J.[4], Kitidis V.[2], McNeill C.L.[2], Ingels J.[6], Parker R[1], Silburn B[1]., Silva T.[1], Sivyer D. B.[1], Smith H.[4], Widdicombe S.[2], Woodward E.M.S.[2], van der Molen J.[1], Garcia L.[1], Kroeger S.[1]

Online Resource 3: Additional plot of observed nutrient fluxes.

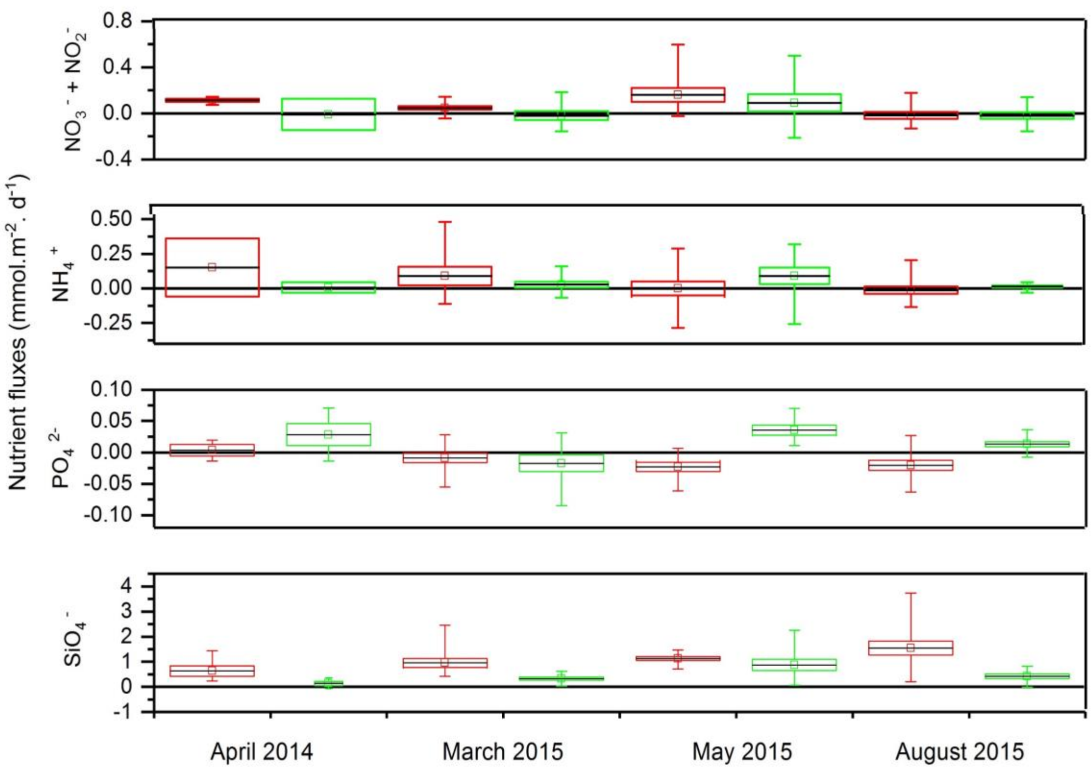

Figure A: Fluxes at site A (red) and G (green). Boxes indicate mean and standard error (5 replicates). Vertical bars are maximum and minimum of the replicates. Derived by monitoring changes in overlying water concentrations from sub-sampled box cores. Value represent combinations of changes in nutrient concentrations due to exchange with bed (the benthic-pelagic flux) and processes within the overlying water.
