# Supplementary material for: Comparing benthic biogeochemistry at a sandy and a muddy site in the Celtic Sea using a model and observations
Source: Biogeochemistry. 2017 Sep 7;135(1):155–82. doi: 10.1007/s10533-017-0367-0 (PMC6961523; doi:10.1007/s10533-017-0367-0)
Supplement: Supplementary file 4 — Supplementary material 4 (PDF 289 kb) [file 10533_2017_367_MOESM4_ESM.pdf]

Comparing benthic biogeochemistry at a sandy and a muddy site in the Celtic Sea using a model and observations

Aldridge J.N.* [1], Lessin G.[2], Amoudry L. O.[5], Hicks N.[3], Hull T.[1], Klar J.[4], Kitidis V.[2], McNeill C.L.[2], Ingels J.[6], Parker R[1,] Silburn B[1]., Silva T.[1], Sivyer D. B.[1], Smith H.[4,] Widdicombe S.[2], Woodward E.M.S.[2], van der Molen J.[1], Garcia L.[1], Kroeger S.[1]

Online Resource 4: Condensed summary and synthesis of model data comparisons. Greyscale of each box indicates subjective assessment of level of agreement. White = good agreement, light grey = moderate agreement, dark grey=poor agreement

Quantity	Model A	Model A1	Model G
Surface temperature	Modelled surface temperatures were in good agreement in spring and autumn and overestimated in July and August.	see Model A	see Model A
Bottom temperature	Modelled bottom temperatures were successfully relaxed to observed values. Good reproduction of the onset of stratification in 2014 and 2015.	see Model A	see Model A
Surface chlorophyll	Timing and magnitude of spring bloom well represented. Underestimate (up to a factor of 2) in modelled chlorophyll after the spring bloom. Model suggests that production has moved to the thermocline, so surface chlorophyll may not be strong indicator of production (see discussion section 5.1)	see Model A	see Model A
Surface oxygen	Good agreement apart from an underestimate of winter values at the start of 2015. Observed peak surface-oxygen concentrations associated with spring bloom qualitatively reproduced by the model.	see Model A	see Model A
Bottom oxygen	Bottom oxygen concentrations after the onset of stratification decreased less rapidly than observed. A ~10% overestimate	see Model A	see Model A

	just before water-column remixing.		
Pelagic nitrate, phosphate, silicate concentrations	Nitrate, phosphate, silicate in good agreement with measurements.	see Model A	see Model A
Pelagic ammonium concentrations	Poor agreement in 2014, better agreement in 2015. Observed bottom value showed evidence of decrease over summer 2015 while model values remained constant.	see Model A	see Model A
Benthic particulate organic<br>carbon (POC) and nitrogen	Model values approximately 1% of observed. The model pool is primarily the biologically available material. It is suggested that the discrepancy is due to observed POC being composed primarily of biologically inactive material.	No substantive difference to Model A result.	No substantive difference to Model A result.
Benthic oxygen uptake (TOU)	Model within range of observed values	Model close to middle of range of observed value.	model within range of observed values
Oxygen penetration depth (OPD)	Model overestimates prior and during spring bloom on average by 1 cm (observed average ~1cm verses model average ~2cm). Good agreement in August 2015.	Results closer to observed values than model A. Overestimated compared to May 2015 observations (observed ~0.5cm verses model ~1cm)	Captured overall change from 5cm to 1 cm pre- and post-bloom, but detailed timing of changes was poor. Observed inter-annual variability not captured by the model.
Pore water nitrate concentrations	Concentration in winter and spring became extremely high compared with observations due to reduced nitrate removal to N2. This occurred when bacterial biomass became small due to reduced benthic organic matter concentration prior to the spring bloom. Better agreement with August observations due to increase as nitrate removal rates.	Better agreement with observations prior and immediately after the spring bloom compared to model A. This was due to sustained nitrate removal through the annual cycle due to more increased availability of particulate organic matter in this parameterisation.	Concentration in winter and spring became extremely high compared with observations. Increased diffusivity to represent pore water flows exacerbated effect of low nitrate removal described for Model A. Better agreement with August observations due to increased nitrate removal rates.

Porewater ammonium, phosphate, silicate concentrations	Concentrations 5%-20% of observed values. Depth average concentrations in the model were broadly similar to observed surface values and the discrepancy appears to be due to an underestimate of concentrations deeper in the sediment.	Re-parameterisation increased ammonium and phosphate and decreased silicate concentrations, although values are still substantially less than observed.	Reasonable agreement at site G. Runs with and without permeable sediment modification had minor effect with respect to agreement with observations
Macrofaunal biomass	Overestimated by factor of 5-10.	Modification to deposit and suspension feeder mortality rates gave total macrofauna biomass values close to observations.	Overestimated by factor of 10-20.
Meiofaunal biomass	Good agreement with observations. However, limited data with measurements only available for late winter/early spring.	The decrease in deposit and suspension feeder biomass reduced grazing pressure on meiofauna leading to a factor two increase in biomass and approximate factor of two overestimate in meiofauna biomass.	Observed meiofauna at site G about half that at A. Model value at G are very similar to A and overestimated observations by a factor of two.
Bacterial biomass	Underestimated by factor of 10. However, observations measure total, not active, bacterial biomass, while model values represent active bacteria.	Underestimated by factor of 10. Re-parameterisation had only a small effect on aerobic bacterial biomass.	Underestimated by factor of 3-5.
